# Experimental materials comparing individual performance implications of two decision aids: Taxonomy and tags

**DOI:** 10.1016/j.mex.2020.101133

**Published:** 2020-11-17

**Authors:** Xuanhui Liu, Karl Werder, Alexander Maedche

**Affiliations:** a Zhejiang Key Laboratory of Design and Intelligence and Digital Creativity, College of Computer Science and Technology, Zhejiang University, China; b Institute of Information Systems and Marketing, Karlsruhe Institute of Technology, Germany; c Cologne Institute for Information Systems, University of Cologne, Germany

**Keywords:** Cognitive effort, Decision aid, Decision style, Decision support, Novice, Tags, Taxonomy

## Abstract

Design techniques have been classified to support the selection in design processes. Two decision aids have been created. We designed an experiment to compare both decision aids (taxonomy and tags) and evaluate the influence of individuals’ decision style when using a decision aid. The experiment materials included the experimental process, a training, an experimental task, and the survey questionnaire. In this method article, we describe the details of the experiment settings and use the collected data to validate the experiment. Advantages of this method include the following:

• The procedure of the experiment ensured an easy-to-understand training part without any bias toward performing the experimental task and answering the survey questionnaire at the end.
• The experimental process can be applied to experiments for evaluating task performance by using user interfaces with a training part before the experimental task.
• The experimental task scenario and the design techniques included in the experiment can be applied in experiments with design-relevant task scenarios.

## Graphical abstract

**Figure:**
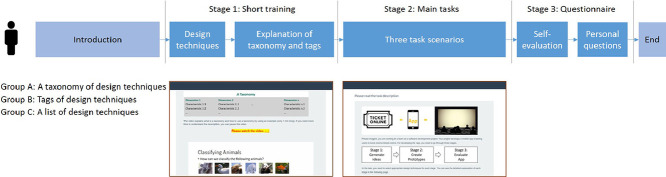

Specifications table

**Table:** 

Subject Area	Computer Science
More specific subject area	*Information Systems*
Method name	*Comparing Individual Performance Implications of Two Decision Aids: Taxonomy and Tags*
Name and reference of original method	*T.M. Shaft, I. Vessey, The Role of Cognitive Fit in the Relationship between Software Comprehension and Modification, MIS Q. 30 (2006) 29–55.* *X. Liu, K. Werder, Q. Zhao, The Role of Cultural Differences when Using Different Classifications: An Experiment Design for Design Technique Selection, in: Proceedings of the 39th International Conference on Information Systems (ICIS), San Francisco, 2018: pp. 1–9.*
Resource availability	*The design techniques used in the experiment and their description, the creation of the design technique decision aids, and the development of the task scenario are available.*

## Method details

The designed experiment compared task performance by using two different decision aids (taxonomy and tags) while accounting for individuals’ decision styles and cognitive efforts. The experiment was based on experimental design to evaluate cognitive fit theory [1]. A prior version of the method, adjusting the experimental method to include decision aids, involved designing an online experiment [2]. This method article describes a laboratory experiment to control for participants’ environmental setting and ensure participants took part in the experiment without interruptions. The study followed a between-subject design, whereas the three groups differed in their decision aids. Next to taxonomy and tags, a list of design techniques was presented to participants in the control group. Each participant was randomly assigned to one of the groups and could only participate once. The experiment was conducted in a well-facilitated computer-based laboratory^1^ with individual soundproofed cabins to ensure that each participant had no interruption from other participants and experimenters during participation. There was also a control room for the experimenters to observe each participant's screen during the experiment and see if the system ran well and whether the participant came across any questions on it. In the following section, we present our experimental materials in further depth by describing participants, procedure, manipulation, measurements, and method validation.

### Participants

For the experiment, 195 participants were invited through a system^2^ based on ORSEE [3]. Twelve sessions were conducted in total (four sessions each day). Each session was randomly assigned to use taxonomy, tags, or a list of design techniques. The experiment was scheduled to last 60 min. The schedule consisted of 60-min breaks between two sessions so that participants could finish the experiment at their own pace, and researchers could sum up the last session and prepare the next one. Each session included at most 20 participants. All of them were asked to arrive at the laboratory's reception 15 min before the experiment started, following the facility's guidelines. The first two authors of this paper organized the 12 sessions together. Each session started with a joint welcome of all participants and a 15-min introduction to the experiment. The introduction included nothing about the experiment content but explained the experimental process, objectives, and reimbursement. Participants received a basic payment for 8 euro for the completion of the experiment and could receive an additional 1 to 3 euro, depending on their performance. If a participant did not finish the experiment task, we paid him or her 5 Euro as a show-up fee. Subsequently, two experimenters guided the participants to their individual cubicles. The main experiment included three stages, as described in the following section.

### Procedure

Table. 1 shows the whole process of the experiment. In the training part (stage 1), we introduced the definitions, the tools used in the task, and the questionnaire in which participants were going to evaluate themselves and answer personal questions; the three task scenarios (stage 2) were the same for all participants. The groups differed in how they could search and select design techniques; specifically, each group had a different decision aid to support their search and selection process. At the end (stage 3), participants answered a questionnaire.

**Table 1:** Experiment process.

Phases	Taxonomy of design techniques	Tags of design techniques	A list of design techniques
**Introduction**	• Data privacy • Short overview of the three stages		
**Stage 1**	**Knowledge of design techniques** • The definition of design technique and when to use a design technique A control question on whether the participant understood the explanation of design techniques. • Self-evaluation of previous knowledge of design techniques		
	• What is a taxonomy? • A short video on how to create a taxonomy to classify animals • A short video on how to use the created taxonomy to help with finding animals • Two control questions on whether the participant understood the explanation of taxonomy and the tool.	• What are tags? • A short video on how to create tags to classify animals • A short video on how to use the created tags to help with finding animals • Two control questions on whether the participant understood the explanation of tags and the tool.	• A short video on how to use a list to find animals • A control question on whether the participant understood the explanation of the tool.
**Stage 2**	**Three task scenarios** • Overview of the whole task (no detailed information on each task scenario) • A mobile app development context including three design scenarios to select design techniques. • Selecting techniques for the 1st task scenario • A control question on whether the participant understood the explanation of the selection task • One-minute washout phase • Selecting techniques for the 2nd task scenario • A control question on whether the participant understood the explanation of the selection task • One-minute washout phase • Selecting techniques for the 3rd task scenario • A control question on whether the participant understood the explanation of the selection task		
	• The tool for using a taxonomy of design techniques for the selection task	• The tool for using tags of design techniques for the selection task	• The tool for using a list of design techniques for the selection task
**Stage 3**	**Self-evaluation and Personal questions** • Cognitive effort and decision styles (intuitive or rational) • Age, gender, education		
**Payment**	• Experimenters checked the correctness of each participant's answer based on the predefined correct answers to the experiment tasks • Experimenters paid each participant based on the correctness		

The participants, at first, attended the introduction of the experiment, where we introduced data privacy and the three stages of the experiment. Participants were informed that the data would be analyzed as a whole, and no one had access to the exact individual data collected in the experiment. Then, the researchers introduced the three stages to the participants, who needed around 45 min to finish the entire experiment.

In stage 1, the participants could review the meaning of the design techniques, taxonomy, and tags to understand the application of design techniques in digital service design processes and get familiar with the experiment tool that they would use in the following tasks. After introducing the definition of design techniques, we asked some questions on prior knowledge of design techniques. We asked questions directly after the description of design techniques instead of after finishing the tasks to make sure that the implementation of selection tasks would not influence the self-evaluation of prior knowledge.

**Fig. 1:**
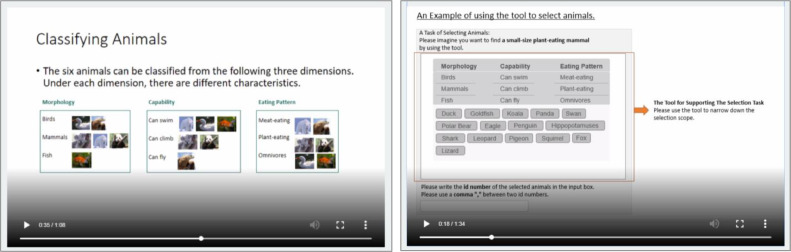
Screenshots of the description of creating a taxonomy (left) and the description of using a taxonomy (right).

**Fig. 2:**
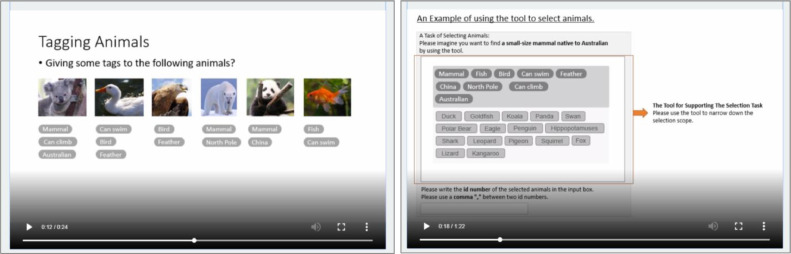
Screenshots of the description of creating tags (left) and the description of using tags (right).

We gave no hint to the tasks to make sure that the training part would not bias participants. Instead of explaining the definition of taxonomy and tags, we used an example of classifying animals in training. The participants viewed a short video to describe how animals could be classified into structured categories (i.e., taxonomy) or unstructured categories (i.e., tags). The short videos to describe the tool for the experiment task applied the created taxonomy and tags of animals for filtering animals. The videos introduced participants to the tasks, explaining how they could use the categories to filter information, where they could find the detailed description of each entry, and where they had to type in their selections. For the control group, there was no description of the classification. The introduction of the experiment tool used an example of selecting animals from a list. Fig. 1 illustrates how to use animal classifications to describe the creation of a taxonomy and how to use the created taxonomy. Fig. 2 shows using tagging animals to describe the creation of tags and how to use them. The following control questions were asked to ensure that participants understood the short videos: “Do you think you understand how a taxonomy/tags work when searching for information?” and “Do you think you understand how to use a taxonomy/tags/list to select information for a specific purpose?”

Stage 2 was the experiment part. Each participant was asked to use the provided tool to complete the task. Each stage was a task scenario. Participants were asked to select five design techniques for each scenario. The three scenarios were not presented to participants at one time but were separated. The next task scenario would be presented after participants provided answers to the first task scenario and answered a control question on whether they understood the scenario (“Do you think you understand the task description?”). Participants then watched a natural picture for one minute as a washout phase. We designed the task process to make sure participants concentrated on only one scenario at a time and were not influenced by other scenarios. As participants were design novices who had limited knowledge of design techniques, we gave those who were assigned to the two treatment groups a piece of paper that explained the meaning of the categories included in the taxonomy or tags.

After finishing the task, participants went to stage 3, where they were asked to answer a questionnaire on measurements and demographic data. First, participants answered a question on cognitive effort spent on the tasks. Then, they answered questions on their decision style (i.e., intuitive or rational). Finally, they provided their demographical information. We designed the questionnaire to first show questions on cognitive effort because these questions needed participants to recall their task completion process. When participants finished the experiment tasks and the questionnaire, they were informed that they could leave the cabin and go to the receptionist.

At the reception, we checked the answers’ correctness and gave money to the participants based on answers’ accuracy. We had predefined five correct selections for each task scenario: if a participants’ answers included four out of these five correct selections, we gave an extra 1 euro for the good performance. Each participant could at most receive 3 euro extra. The basic payment was 8 euro. If a participant did not finish the whole experiment, he or she received 5 euro. The payment amount varied between 8 and 11. Participants were not allowed to go back to the experiment room to prevent them from interrupting other participants who had not finished yet.

### Manipulation

The three task scenarios in stage 2 were synthesized by reviewing 31 scenarios of using design techniques in previous studies (Table 2). We predefined correct answers for each task scenario.

**Table 2:** Synthesizing task scenarios.

Design technique	Dimension 1	Dimension 2	Dimension 3	Study
3-12-3 Brainstorming	Idea generation, rough ideas	30 min	10 people	[4]
3E- Expressing Emotion and Experience	Understand emotion, feelings	5 days, need time and effort	Real users	[5]
6-3-5 Brainwriting	Idea generation, rough ideas	30 min	6 people	[6]
A/B Testing	Live website, test version, ready to release version	Long-term or short-term	Real users	[7]
Bodystorming	Evaluate draft prototype	Short-term	Designers	[8]
Business Origami	Idea, analyze relations	Fast	Around 10 people	[9]
Co-discovery	Observe users when using a product	Short-term	Two users	[10]
Collaborative Sketching	Prototyping	Short-term	Designers, stakeholders	[11]
Concurrent Think-aloud	Live Web	A couple of days	Users	[12]
Desirability Testing with Product Research Cards	Compare prototypes	A couple of hours	Users	[13]
Diary Studies	Live App	Long-term	Users	[14]
Experience Clip	Live Web	A couple of days	Users	[15]
Experience Prototyping	Evaluate draft prototype	Short-term	Designers	[8]
Eye-tracking	Live Web	Short-term	Users	[16]
Flexible Modeling	Prototyping	Short-term	Users	[17]
Heuristic Evaluation	Live web	Short-term	Experts	[18]
Mood Boards	Ideas	Short-term	Design participants	[19]
Offering Map	Detail a product/service, Idea generation,	Short-term	Discussion	[20]
Parallel Prototyping	Prototyping	Long-term	A group of designers	[21]
Private Camera Conversation	Detailed prototype or a product	A couple of hours	User	[22]
Product Experience Tracker	Live Web	A couple of days	Users	[23]
Repertory Grid Technique	Prototyping evaluation	A couple of hours	Users	[24]
Retrospective Think-aloud	Live Web	A couple of days	Users	[25]
Role-playing	Ideas	A couple of hours	A group of people	[26]
Speed Dating	Prototype evaluation	A couple of hours	Users	[27]
Storyboarding	Early-stage, show interaction between user and product/service	Short-term	Discussion	[28]
Story Sharing	Ideas	A couple of hours	A group of people	[29]
Tomorrow Headlines	Shared vision for future	A couple of days	Multidisciplinary team	[18]
UX Curve	Live Web	Long-term	User	[30]
Wireframe	Prototypes	A couple of days	A group of designers	[31]
Wizard of Oz	Prototypes	Short-term	A group of designers	[32]

The developed task description is as follows.

Please imagine you are working in a team on a project. Your project develops a mobile app enabling users to book cinema tickets. Please select suitable design techniques based on three design stages:

• Stage 1: Generate ideas
  ○ You are at an early stage of your project.
  ○ You want to involve a group of people (more than two) to discuss the project and generate some ideas during the discussion.
  ○ You do not have access to real users.
• Stage 2: Create prototypes
  ○ You now want to create some initial prototypes based on the ideas created in stage 1.
  ○ After creating several prototypes, you want to compare them and decide on one or two prototypes for further refinement.
  ○ You again do not have access to real users when creating and evaluating prototypes.
• Stage 3: Evaluate App
  ○ You now have developed a first running version of the app
  ○ You want to evaluate the app with real users before delivering it on a large scale to the market
  ○ The evaluation seeks to collect real users' data and feedback within a couple of days.

### Measurements

The measurements we used in the experiment included prior design technique knowledge, intuitive decision style, rational decision style, and cognitive effort, which were measured by a 7-point Likert scale (1 = strongly disagree; 7 = strongly agree). Participants were asked to evaluate the degree of agreement to the items included in each measurement. The items for prior design technique knowledge directly appeared after the explanation of the design techniques in stage 1. The questions for measuring the other three variables were asked in stage 3. Table 3 presents the measurements and the items.

**Table 3:** Measurements and items.

Measurement	Item
Rational decision styles [33]	• I prefer to gather all the necessary information before committing to a decision. • I thoroughly evaluate decision alternatives before making a final choice. • In decision-making, I take time to contemplate the pros/cons or risks/benefits of a situation. • Investigating the facts is an important part of my decision-making process. • I weigh a number of different factors when making decisions.
Intuitive decision styles [33]	• When making decisions, I rely mainly on my gut feelings. • My initial hunch about decisions is generally what I follow. • I make decisions based on intuition. • I rely on my first impressions when making decisions. • I weigh feelings more than analysis in making decisions.
Cognitive effort [34]	• To complete the task, using [the taxonomy/tags/list] was very frustrating. • To complete the task, using [the taxonomy/tags/list] took too much time. • To complete the task, using [the taxonomy/tags/list] required too much effort. • To complete the task, using [the taxonomy/tags/list] was too complex. • To complete the task, using [the taxonomy/tags/list] was easy.
Prior design technique knowledge [35]	• I know pretty much about design techniques. • In a design group, I am one of the experts when using design techniques. • Compare to most other members in a design team, I know less about these products. • When using a design technique, I really do not know a lot. • I do not feel very knowledgeable about design techniques. • I have a lot of experiences with design techniques. • I feel familiar with design techniques.

### Method validation

195 subjects participated in the experiment. Two of them did not understand some parts of the experiment. We filtered them out based on the “No” answer to the control questions. Three of them did not finish the whole experiment. 190 participants provided valid data. 97.4% of the whole data points from the experiment were valid, which also meant the experiment was designed in an easy-to-understand way.

The Cronbach's alpha of prior design technique knowledge, rational decision style, intuitive decision style, and cognitive effort were 0.91, 0.86, 0.88, and 0.89, respectively. Furthermore, the items loaded highly on their assigned factors and lowly on other factors. The validation checks showed adequate reliability of the measurement scales.

**Fig. 3:**
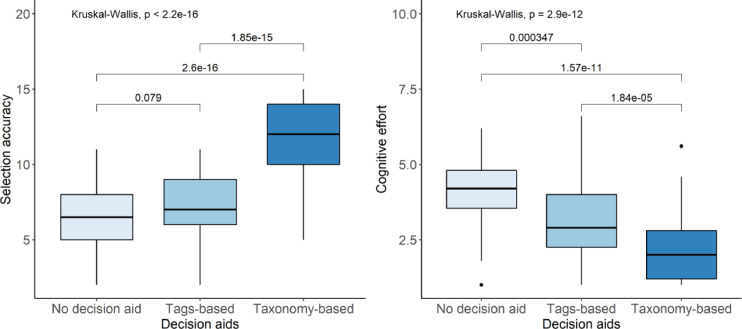
The effect of decision aids on selection accuracy (left) and cognitive effort (right). The *p* value from Mann-Whitney U test shows between two groups also presented on both plots.

The results of the comparison of the three groups of participants (64 for the taxonomy, 62 for the tags, 64 for the list) indicated a statistically significant difference by using the Kruskal-Wallis test and the Mann-Whitney U test (Fig. 3).

## Declaration of Competing Interest

The authors declare that they have no known competing financial interests or personal relationships that could have appeared to influence the work reported in this paper.

## Appendix Supplementary materials

Image, application 1

Image, application 2

Image, application 3

Image, application 4

Image, video 5

Image, video 6

Image, video 7

Image, video 8

Image, video 9

## Footnotes

1 The website of KD2 Lab is http://www.kd2lab.kit.edu/english/index.php.

2 https://iism-kd2-hroot.iism.kit.edu/?locale=en.
