# Supplementary material for: Experimental materials comparing individual performance implications of two decision aids: Taxonomy and tags
Source: MethodsX. 2020 Nov 17;7:101133. doi: 10.1016/j.mex.2020.101133 (PMC7701260; doi:10.1016/j.mex.2020.101133)
Supplement: Supplementary file 1 [file mmc1.zip › Data-Analysis-R.html]

Data Analysis


# Data Analysis

#### 2020/5/4

### Description

##### COG\_EFF = Cognitive effort

##### DES\_AIS\_RA = Rational decision style

##### DES\_AIS\_IN = Intuitive decision style

##### CORRECT\_ALL = Selection accuracy

##### CLASS = 2: taxonomy-based decision aid; 1: tags-based decision aid; 0: no decision aid

```
# packages
library(ggplot2)
library(magrittr)
library(ggpubr)
library(Hmisc)
```

```
## Loading required package: lattice
```

```
## Loading required package: survival
```

```
## Warning: package 'survival' was built under R version 3.6.3
```

```
## Loading required package: Formula
```

```
## 
## Attaching package: 'Hmisc'
```

```
## The following objects are masked from 'package:base':
## 
##     format.pval, units
```

```
library(ggsci)
library(ggsignif)
library(carData)
library(car)
library(psych)
```

```
## 
## Attaching package: 'psych'
```

```
## The following object is masked from 'package:car':
## 
##     logit
```

```
## The following object is masked from 'package:Hmisc':
## 
##     describe
```

```
## The following objects are masked from 'package:ggplot2':
## 
##     %+%, alpha
```

```
library(MASS)
library(Matrix)
library(mvtnorm)
library(sandwich)
library(lavaan)
```

```
## This is lavaan 0.6-5
```

```
## lavaan is BETA software! Please report any bugs.
```

```
## 
## Attaching package: 'lavaan'
```

```
## The following object is masked from 'package:psych':
## 
##     cor2cov
```

```
library(semTools)
```

```
##
```

```
## ###############################################################################
```

```
## This is semTools 0.5-2
```

```
## All users of R (or SEM) are invited to submit functions or ideas for functions.
```

```
## ###############################################################################
```

```
## 
## Attaching package: 'semTools'
```

```
## The following object is masked from 'package:psych':
## 
##     skew
```

```
library(betas)
library(MBESS)
```

```
## 
## Attaching package: 'MBESS'
```

```
## The following object is masked from 'package:lavaan':
## 
##     cor2cov
```

```
## The following object is masked from 'package:psych':
## 
##     cor2cov
```

```
library(mediation)
```

```
## Registered S3 methods overwritten by 'lme4':
##   method                          from
##   cooks.distance.influence.merMod car 
##   influence.merMod                car 
##   dfbeta.influence.merMod         car 
##   dfbetas.influence.merMod        car
```

```
## mediation: Causal Mediation Analysis
## Version: 4.5.0
```

```
## 
## Attaching package: 'mediation'
```

```
## The following object is masked from 'package:psych':
## 
##     mediate
```

```
library(multilevel)
```

```
## Loading required package: nlme
```

```
library(stargazer)
```

```
## 
## Please cite as:
```

```
##  Hlavac, Marek (2018). stargazer: Well-Formatted Regression and Summary Statistics Tables.
```

```
##  R package version 5.2.2. https://CRAN.R-project.org/package=stargazer
```

```
library(doBy)
library(boot)
```

```
## 
## Attaching package: 'boot'
```

```
## The following object is masked from 'package:psych':
## 
##     logit
```

```
## The following object is masked from 'package:car':
## 
##     logit
```

```
## The following object is masked from 'package:survival':
## 
##     aml
```

```
## The following object is masked from 'package:lattice':
## 
##     melanoma
```

```
library(plyr)
```

```
## 
## Attaching package: 'plyr'
```

```
## The following objects are masked from 'package:Hmisc':
## 
##     is.discrete, summarize
```

```
## The following object is masked from 'package:ggpubr':
## 
##     mutate
```

```
library(rstatix)
```

```
## Warning: package 'rstatix' was built under R version 3.6.3
```

```
## 
## Attaching package: 'rstatix'
```

```
## The following objects are masked from 'package:plyr':
## 
##     desc, mutate
```

```
## The following object is masked from 'package:MASS':
## 
##     select
```

```
## The following object is masked from 'package:stats':
## 
##     filter
```

```
# Read data
Experiment <- read.csv(file = "C:/Data/Experiment Data Final.csv", header = TRUE)

# Compare there is no difference between student group and other
Experiment$JOB <- Experiment$What.is.your.job.status.
Experiment$JOB <- revalue(Experiment$JOB, c("Freelancer" = "other", 
                                            "without Employee" = "other",
                                            "unemployed" = "other", 
                                            "Employee" = "other"))
Experiment$JOB
```

```
##   [1] Student Student Student Student Student Student Student Student
##   [9] Student Student Student Student Student Student Student Student
##  [17] Student Student Student Student Student Student Student Student
##  [25] Student Student Student Student Student Student Student Student
##  [33] Student Student Student Student Student Student Student Student
##  [41] Student Student Student Student Student Student Student Student
##  [49] Student Student Student Student Student Student Student Student
##  [57] Student Student Student Student Student other   Student Student
##  [65] Student other   Student Student Student Student Student Student
##  [73] Student Student Student Student Student Student Student Student
##  [81] Student Student Student Student Student Student Student Student
##  [89] other   Student Student Student Student Student Student Student
##  [97] Student Student Student Student Student Student other   Student
## [105] Student Student Student Student Student Student Student Student
## [113] Student Student Student Student Student Student Student Student
## [121] Student Student Student Student Student Student Student Student
## [129] Student Student Student Student Student Student Student Student
## [137] Student other   Student Student Student Student Student Student
## [145] Student Student Student Student Student Student Student Student
## [153] Student Student Student Student Student Student Student Student
## [161] Student Student other   Student Student Student Student Student
## [169] Student Student Student Student Student Student Student Student
## [177] Student Student Student Student Student Student Student Student
## [185] Student Student Student Student Student Student
## Levels: other Student
```

```
Experiment$JOB <- as.factor(Experiment$JOB)
wilcox.test(CORRECT_ALL ~ JOB, data = Experiment, paired = FALSE)
```

```
## 
##  Wilcoxon rank sum test with continuity correction
## 
## data:  CORRECT_ALL by JOB
## W = 607, p-value = 0.6793
## alternative hypothesis: true location shift is not equal to 0
```

```
wilcox.test(COG_EFF ~ JOB, data = Experiment, paired = FALSE)
```

```
## 
##  Wilcoxon rank sum test with continuity correction
## 
## data:  COG_EFF by JOB
## W = 578.5, p-value = 0.8443
## alternative hypothesis: true location shift is not equal to 0
```

```
# CLASS as factor
Experiment$CLASS <- as.factor(Experiment$CLASS)
# Creat subset of the data
attach(Experiment)
# Subset with taxonomy and control
ExperimentDataTAXO <- subset(Experiment, CLASS == "2" | CLASS == "0")
# Subset with tags and control
ExperimentDataTAGS <- subset(Experiment, CLASS == "1" | CLASS == "0")
# Subset with taxonomy and tags
ExperimentDataTAXOTAGS <- subset(Experiment, CLASS == "2" | CLASS == "1")
```

### Measurement Validity

```
# Cronbach alphas of prior design technique knowledge
alpha(Experiment[,5:11])
```

```
## 
## Reliability analysis   
## Call: alpha(x = Experiment[, 5:11])
## 
##   raw_alpha std.alpha G6(smc) average_r S/N    ase mean  sd median_r
##       0.91      0.91    0.91      0.61  11 0.0098  2.9 1.2     0.62
## 
##  lower alpha upper     95% confidence boundaries
## 0.89 0.91 0.93 
## 
##  Reliability if an item is dropped:
##           raw_alpha std.alpha G6(smc) average_r  S/N alpha se  var.r med.r
## TECH_KN01      0.89      0.89    0.89      0.58  8.3   0.0124 0.0084  0.60
## TECH_KN02      0.89      0.89    0.89      0.58  8.3   0.0124 0.0091  0.59
## TECH_KN03      0.91      0.91    0.90      0.62 10.0   0.0107 0.0097  0.63
## TECH_KN04      0.91      0.91    0.91      0.64 10.6   0.0099 0.0082  0.64
## TECH_KN05      0.89      0.90    0.89      0.59  8.5   0.0125 0.0112  0.59
## TECH_KN06      0.90      0.91    0.90      0.62  9.9   0.0108 0.0088  0.62
## TECH_KN07      0.90      0.90    0.89      0.61  9.2   0.0114 0.0105  0.60
## 
##  Item statistics 
##             n raw.r std.r r.cor r.drop mean  sd
## TECH_KN01 190  0.87  0.88  0.87   0.82  2.9 1.4
## TECH_KN02 190  0.87  0.88  0.87   0.83  2.5 1.3
## TECH_KN03 190  0.77  0.76  0.71   0.68  3.1 1.6
## TECH_KN04 190  0.74  0.73  0.66   0.63  3.6 1.6
## TECH_KN05 190  0.87  0.86  0.84   0.80  3.1 1.6
## TECH_KN06 190  0.76  0.77  0.72   0.68  2.2 1.3
## TECH_KN07 190  0.81  0.82  0.78   0.74  2.8 1.3
## 
## Non missing response frequency for each item
##              1    2    3    4    5    6    7 miss
## TECH_KN01 0.13 0.38 0.21 0.09 0.15 0.03 0.01    0
## TECH_KN02 0.23 0.43 0.13 0.12 0.07 0.02 0.01    0
## TECH_KN03 0.11 0.35 0.19 0.12 0.12 0.10 0.01    0
## TECH_KN04 0.06 0.26 0.22 0.13 0.16 0.15 0.03    0
## TECH_KN05 0.10 0.39 0.21 0.07 0.11 0.09 0.02    0
## TECH_KN06 0.28 0.47 0.09 0.06 0.05 0.03 0.01    0
## TECH_KN07 0.14 0.39 0.22 0.10 0.11 0.04 0.00    0
```

```
# Cronbach alphas of system cognitive effort
alpha(Experiment[,12:16])
```

```
## 
## Reliability analysis   
## Call: alpha(x = Experiment[, 12:16])
## 
##   raw_alpha std.alpha G6(smc) average_r S/N   ase mean  sd median_r
##       0.89      0.89    0.88      0.62 8.1 0.013  3.2 1.4     0.62
## 
##  lower alpha upper     95% confidence boundaries
## 0.86 0.89 0.91 
## 
##  Reliability if an item is dropped:
##           raw_alpha std.alpha G6(smc) average_r S/N alpha se  var.r med.r
## COG_EFF01      0.88      0.89    0.87      0.66 7.9    0.014 0.0067  0.66
## COG_EFF02      0.86      0.86    0.84      0.61 6.2    0.017 0.0105  0.63
## COG_EFF03      0.84      0.85    0.83      0.58 5.5    0.019 0.0089  0.58
## COG_EFF04      0.87      0.87    0.84      0.62 6.4    0.015 0.0110  0.62
## COG_EFF05      0.87      0.87    0.85      0.63 6.8    0.015 0.0079  0.62
## 
##  Item statistics 
##             n raw.r std.r r.cor r.drop mean  sd
## COG_EFF01 190  0.77  0.77  0.68   0.65  3.2 1.7
## COG_EFF02 190  0.87  0.85  0.82   0.77  3.8 1.9
## COG_EFF03 190  0.90  0.89  0.87   0.82  3.2 1.8
## COG_EFF04 190  0.82  0.84  0.79   0.73  2.6 1.5
## COG_EFF05 190  0.81  0.82  0.77   0.70  3.1 1.5
## 
## Non missing response frequency for each item
##              1    2    3    4    5    6    7 miss
## COG_EFF01 0.16 0.31 0.12 0.12 0.19 0.09 0.01    0
## COG_EFF02 0.13 0.22 0.13 0.06 0.21 0.20 0.06    0
## COG_EFF03 0.18 0.30 0.12 0.09 0.14 0.14 0.02    0
## COG_EFF04 0.27 0.36 0.12 0.11 0.11 0.04 0.01    0
## COG_EFF05 0.13 0.31 0.21 0.13 0.13 0.08 0.01    0
```

```
# Cronbach alphas of intuitive decision-making style
alpha(Experiment[,17:21])
```

```
## 
## Reliability analysis   
## Call: alpha(x = Experiment[, 17:21])
## 
##   raw_alpha std.alpha G6(smc) average_r S/N   ase mean  sd median_r
##       0.88      0.88    0.87       0.6 7.5 0.013  3.3 1.1     0.61
## 
##  lower alpha upper     95% confidence boundaries
## 0.86 0.88 0.91 
## 
##  Reliability if an item is dropped:
##             raw_alpha std.alpha G6(smc) average_r S/N alpha se  var.r
## DES_ST_IN01      0.85      0.85    0.81      0.58 5.5    0.018 0.0052
## DES_ST_IN02      0.84      0.84    0.81      0.57 5.3    0.018 0.0069
## DES_ST_IN03      0.86      0.86    0.83      0.60 5.9    0.017 0.0071
## DES_ST_IN04      0.86      0.86    0.83      0.60 6.1    0.016 0.0131
## DES_ST_IN05      0.88      0.88    0.86      0.66 7.6    0.014 0.0034
##             med.r
## DES_ST_IN01  0.58
## DES_ST_IN02  0.57
## DES_ST_IN03  0.58
## DES_ST_IN04  0.61
## DES_ST_IN05  0.64
## 
##  Item statistics 
##               n raw.r std.r r.cor r.drop mean  sd
## DES_ST_IN01 190  0.87  0.86  0.83   0.77  3.3 1.4
## DES_ST_IN02 190  0.87  0.87  0.84   0.79  3.5 1.3
## DES_ST_IN03 190  0.84  0.83  0.78   0.73  3.6 1.3
## DES_ST_IN04 190  0.82  0.82  0.75   0.71  3.4 1.3
## DES_ST_IN05 190  0.74  0.75  0.64   0.60  2.9 1.2
## 
## Non missing response frequency for each item
##                1    2    3    4    5    6    7 miss
## DES_ST_IN01 0.04 0.32 0.24 0.15 0.15 0.09 0.01    0
## DES_ST_IN02 0.02 0.23 0.27 0.21 0.22 0.05 0.00    0
## DES_ST_IN03 0.03 0.21 0.26 0.21 0.21 0.08 0.01    0
## DES_ST_IN04 0.03 0.27 0.27 0.22 0.14 0.06 0.01    0
## DES_ST_IN05 0.09 0.37 0.25 0.17 0.08 0.03 0.00    0
```

```
# Cronbach alphas of rational decision-making style
alpha(Experiment[,22:26])
```

```
## 
## Reliability analysis   
## Call: alpha(x = Experiment[, 22:26])
## 
##   raw_alpha std.alpha G6(smc) average_r S/N   ase mean   sd median_r
##       0.86      0.86    0.84      0.55 6.2 0.016  5.5 0.91     0.55
## 
##  lower alpha upper     95% confidence boundaries
## 0.83 0.86 0.89 
## 
##  Reliability if an item is dropped:
##             raw_alpha std.alpha G6(smc) average_r S/N alpha se  var.r
## DES_ST_RA01      0.83      0.83    0.79      0.54 4.7    0.021 0.0041
## DES_ST_RA02      0.82      0.82    0.78      0.53 4.6    0.021 0.0030
## DES_ST_RA03      0.83      0.83    0.80      0.54 4.8    0.021 0.0080
## DES_ST_RA04      0.83      0.83    0.80      0.55 5.0    0.019 0.0103
## DES_ST_RA05      0.85      0.85    0.82      0.59 5.8    0.017 0.0041
##             med.r
## DES_ST_RA01  0.53
## DES_ST_RA02  0.55
## DES_ST_RA03  0.55
## DES_ST_RA04  0.54
## DES_ST_RA05  0.58
## 
##  Item statistics 
##               n raw.r std.r r.cor r.drop mean  sd
## DES_ST_RA01 190  0.82  0.82  0.77   0.71  5.5 1.2
## DES_ST_RA02 190  0.84  0.83  0.79   0.73  5.4 1.2
## DES_ST_RA03 190  0.82  0.82  0.75   0.70  5.3 1.2
## DES_ST_RA04 190  0.79  0.80  0.73   0.67  5.5 1.0
## DES_ST_RA05 190  0.73  0.74  0.64   0.59  5.5 1.1
## 
## Non missing response frequency for each item
##                1    2    3    4    5    6    7 miss
## DES_ST_RA01 0.00 0.02 0.06 0.06 0.24 0.45 0.17    0
## DES_ST_RA02 0.01 0.02 0.06 0.07 0.27 0.45 0.12    0
## DES_ST_RA03 0.00 0.03 0.06 0.11 0.27 0.39 0.14    0
## DES_ST_RA04 0.00 0.02 0.03 0.10 0.24 0.48 0.13    0
## DES_ST_RA05 0.00 0.02 0.03 0.09 0.29 0.43 0.14    0
```

```
# EFA
# Calculate the correlation matrix
corMat <- cor(Experiment[, c(5:26)])
# Conduct varimax principal component factoring
solution <- principal(r = corMat, nfactors = 4, rotate = "varimax")
# Display the solution output
solution
```

```
## Principal Components Analysis
## Call: principal(r = corMat, nfactors = 4, rotate = "varimax")
## Standardized loadings (pattern matrix) based upon correlation matrix
##               RC2   RC3   RC1   RC4   h2   u2 com
## TECH_KN01    0.87  0.00  0.06  0.07 0.77 0.23 1.0
## TECH_KN02    0.88  0.06  0.14  0.04 0.79 0.21 1.1
## TECH_KN03    0.76  0.03  0.06 -0.04 0.58 0.42 1.0
## TECH_KN04    0.72 -0.06  0.03 -0.11 0.53 0.47 1.1
## TECH_KN05    0.87  0.00  0.02  0.03 0.75 0.25 1.0
## TECH_KN06    0.77 -0.05  0.03  0.10 0.60 0.40 1.0
## TECH_KN07    0.81 -0.08  0.05  0.08 0.67 0.33 1.0
## COG_EFF01   -0.01  0.75  0.00 -0.15 0.58 0.42 1.1
## COG_EFF02   -0.11  0.86  0.00  0.03 0.75 0.25 1.0
## COG_EFF03   -0.04  0.89  0.09 -0.08 0.80 0.20 1.0
## COG_EFF04    0.08  0.83  0.14 -0.07 0.72 0.28 1.1
## COG_EFF05   -0.01  0.82  0.03 -0.03 0.68 0.32 1.0
## DES_ST_IN01  0.03  0.00  0.87 -0.11 0.77 0.23 1.0
## DES_ST_IN02  0.09  0.08  0.86 -0.11 0.77 0.23 1.1
## DES_ST_IN03  0.01 -0.03  0.85 -0.07 0.73 0.27 1.0
## DES_ST_IN04  0.09  0.13  0.79 -0.17 0.67 0.33 1.2
## DES_ST_IN05  0.16  0.11  0.65 -0.31 0.55 0.45 1.6
## DES_ST_RA01  0.11 -0.06 -0.21  0.78 0.68 0.32 1.2
## DES_ST_RA02  0.12 -0.08 -0.10  0.83 0.71 0.29 1.1
## DES_ST_RA03  0.05 -0.02 -0.07  0.82 0.68 0.32 1.0
## DES_ST_RA04  0.01 -0.11 -0.19  0.76 0.63 0.37 1.2
## DES_ST_RA05 -0.12 -0.03 -0.12  0.73 0.57 0.43 1.1
## 
##                        RC2  RC3  RC1  RC4
## SS loadings           4.73 3.53 3.43 3.31
## Proportion Var        0.21 0.16 0.16 0.15
## Cumulative Var        0.21 0.38 0.53 0.68
## Proportion Explained  0.32 0.24 0.23 0.22
## Cumulative Proportion 0.32 0.55 0.78 1.00
## 
## Mean item complexity =  1.1
## Test of the hypothesis that 4 components are sufficient.
## 
## The root mean square of the residuals (RMSR) is  0.05 
## 
## Fit based upon off diagonal values = 0.98
```

```
# Mean, Median, SD
# Selection accuracy
mean(Experiment$CORRECT_ALL)
```

```
## [1] 8.589474
```

```
median(Experiment$CORRECT_ALL)
```

```
## [1] 8
```

```
sd(Experiment$CORRECT_ALL)
```

```
## [1] 3.202164
```

```
# System cognitive effort
mean(Experiment$COG_EFF)
```

```
## [1] 3.183158
```

```
median(Experiment$COG_EFF)
```

```
## [1] 3
```

```
sd(Experiment$COG_EFF)
```

```
## [1] 1.405857
```

```
# Rational decision-making style
mean(Experiment$DES_ST_RA)
```

```
## [1] 5.468421
```

```
median(Experiment$DES_ST_RA)
```

```
## [1] 5.7
```

```
sd(Experiment$DES_ST_RA)
```

```
## [1] 0.9062701
```

```
# Intuitive decision-making style
mean(Experiment$DES_ST_IN)
```

```
## [1] 3.348421
```

```
median(Experiment$DES_ST_IN)
```

```
## [1] 3.2
```

```
sd(Experiment$DES_ST_IN)
```

```
## [1] 1.084412
```

```
# Perceived design technique knowledge
mean(Experiment$TECH_KN)
```

```
## [1] 2.874436
```

```
median(Experiment$TECH_KN)
```

```
## [1] 2.571429
```

```
sd(Experiment$TECH_KN)
```

```
## [1] 1.167729
```

```
# Correlation of the variables
corMat <- cor(Experiment[, c(27:31)])
corMat
```

```
##                 TECH_KN     COG_EFF    DES_ST_IN   DES_ST_RA  CORRECT_ALL
## TECH_KN      1.00000000 -0.04172013  0.147546231  0.05787174  0.029804099
## COG_EFF     -0.04172013  1.00000000  0.137834069 -0.15191321 -0.466262564
## DES_ST_IN    0.14754623  0.13783407  1.000000000 -0.34073568 -0.009786981
## DES_ST_RA    0.05787174 -0.15191321 -0.340735679  1.00000000  0.161421335
## CORRECT_ALL  0.02980410 -0.46626256 -0.009786981  0.16142134  1.000000000
```

```
# P value of the correlation
cor_2 <- rcorr(as.matrix(Experiment[, c(27:31)]))
cor_2
```

```
##             TECH_KN COG_EFF DES_ST_IN DES_ST_RA CORRECT_ALL
## TECH_KN        1.00   -0.04      0.15      0.06        0.03
## COG_EFF       -0.04    1.00      0.14     -0.15       -0.47
## DES_ST_IN      0.15    0.14      1.00     -0.34       -0.01
## DES_ST_RA      0.06   -0.15     -0.34      1.00        0.16
## CORRECT_ALL    0.03   -0.47     -0.01      0.16        1.00
## 
## n= 190 
## 
## 
## P
##             TECH_KN COG_EFF DES_ST_IN DES_ST_RA CORRECT_ALL
## TECH_KN             0.5676  0.0422    0.4277    0.6831     
## COG_EFF     0.5676          0.0579    0.0364    0.0000     
## DES_ST_IN   0.0422  0.0579            0.0000    0.8934     
## DES_ST_RA   0.4277  0.0364  0.0000              0.0261     
## CORRECT_ALL 0.6831  0.0000  0.8934    0.0261
```

```
# Calculate square root of AVE of the measurement
HS.model <- '
            COG_EFF =~ COG_EFF01 + COG_EFF02 + COG_EFF03 + COG_EFF04 + COG_EFF05
            DES_ST_IN =~ DES_ST_IN01 + DES_ST_IN02 + DES_ST_IN03 + DES_ST_IN04 + DES_ST_IN05
            DES_ST_RA =~ DES_ST_RA01 + DES_ST_RA02 + DES_ST_RA03 + DES_ST_RA04 + DES_ST_RA05
            TECH_KN =~ TECH_KN01 + TECH_KN02 + TECH_KN03 + TECH_KN04 + TECH_KN05 + TECH_KN06 + TECH_KN07
            '
fit <- cfa(HS.model, data = Experiment, std.lv = TRUE)
ave <- reliability(fit)
sqrt(ave[5,])
```

```
##   COG_EFF DES_ST_IN DES_ST_RA   TECH_KN     total 
## 0.7987047 0.7847469 0.7512786 0.7765898 0.7818554
```

### Hypotheses test

#### H1: Using a taxonomy-based decision aid leads to higher selection accuracy than using a tags-based decision aid, whereas using a tags-based decision aid leads to higher selection accuracy than using no decision aid at all.

```
# Hierarchical linear regression
# The regression of all control variables on selection accuracy
fit11 <- lm(CORRECT_ALL ~ Age + Gender + EDU + TECH_KN, data = Experiment)
summary(fit11)
```

```
## 
## Call:
## lm(formula = CORRECT_ALL ~ Age + Gender + EDU + TECH_KN, data = Experiment)
## 
## Residuals:
##     Min      1Q  Median      3Q     Max 
## -6.7256 -2.4312 -0.3958  2.3044  6.7212 
## 
## Coefficients:
##              Estimate Std. Error t value Pr(>|t|)    
## (Intercept)  8.740856   1.636425   5.341 2.69e-07 ***
## Age          0.009477   0.069702   0.136    0.892    
## Gender      -0.324529   0.521268  -0.623    0.534    
## EDU         -0.094952   0.489846  -0.194    0.847    
## TECH_KN      0.065306   0.204220   0.320    0.749    
## ---
## Signif. codes:  0 '***' 0.001 '**' 0.01 '*' 0.05 '.' 0.1 ' ' 1
## 
## Residual standard error: 3.231 on 185 degrees of freedom
## Multiple R-squared:  0.003279,   Adjusted R-squared:  -0.01827 
## F-statistic: 0.1522 on 4 and 185 DF,  p-value: 0.9618
```

```
# The regrssion of decision aids on selection accuracy
fit12 <- lm(CORRECT_ALL ~ Age + Gender + EDU + TECH_KN + CLASS, data = Experiment)
summary(fit12)
```

```
## 
## Call:
## lm(formula = CORRECT_ALL ~ Age + Gender + EDU + TECH_KN + CLASS, 
##     data = Experiment)
## 
## Residuals:
##    Min     1Q Median     3Q    Max 
## -6.734 -1.448  0.250  1.566  4.432 
## 
## Coefficients:
##             Estimate Std. Error t value Pr(>|t|)    
## (Intercept)  6.94135    1.18918   5.837 2.37e-08 ***
## Age          0.01180    0.04972   0.237   0.8127    
## Gender      -0.02030    0.37243  -0.054   0.9566    
## EDU         -0.20331    0.34951  -0.582   0.5615    
## TECH_KN     -0.09353    0.14617  -0.640   0.5231    
## CLASS1       0.74782    0.41119   1.819   0.0706 .  
## CLASS2       5.11067    0.41017  12.460  < 2e-16 ***
## ---
## Signif. codes:  0 '***' 0.001 '**' 0.01 '*' 0.05 '.' 0.1 ' ' 1
## 
## Residual standard error: 2.304 on 183 degrees of freedom
## Multiple R-squared:  0.4986, Adjusted R-squared:  0.4821 
## F-statistic: 30.33 on 6 and 183 DF,  p-value: < 2.2e-16
```

```
# Robust test of fit12
bootReg <- function (formula, data, indices)
{
  d <- data [indices,]
  fit <- lm(formula, data = d)
  return(coef(fit))
}

bootResults<-boot(statistic = bootReg, formula = CORRECT_ALL ~ Age + Gender + EDU + TECH_KN + CLASS, data = Experiment, R = 2000)
summary(bootResults)
```

```
## 
## Number of bootstrap replications R = 2000 
##    original   bootBias   bootSE   bootMed
## 1  6.941345 -0.1284334 1.095802  6.865706
## 2  0.011797  0.0054244 0.047449  0.013691
## 3 -0.020296 -0.0049486 0.388919 -0.017525
## 4 -0.203305 -0.0065241 0.336373 -0.203858
## 5 -0.093532  0.0015655 0.133969 -0.090277
## 6  0.747820  0.0150357 0.400323  0.759690
## 7  5.110668  0.0189185 0.404940  5.136855
```

```
# confidence intervals in the bootstrapped sample
boot.ci(bootResults, type = "bca", index = 1)
```

```
## BOOTSTRAP CONFIDENCE INTERVAL CALCULATIONS
## Based on 2000 bootstrap replicates
## 
## CALL : 
## boot.ci(boot.out = bootResults, type = "bca", index = 1)
## 
## Intervals : 
## Level       BCa          
## 95%   ( 4.703,  8.995 )  
## Calculations and Intervals on Original Scale
```

```
boot.ci(bootResults, type = "bca", index = 2)
```

```
## BOOTSTRAP CONFIDENCE INTERVAL CALCULATIONS
## Based on 2000 bootstrap replicates
## 
## CALL : 
## boot.ci(boot.out = bootResults, type = "bca", index = 2)
## 
## Intervals : 
## Level       BCa          
## 95%   (-0.0770,  0.1083 )  
## Calculations and Intervals on Original Scale
```

```
boot.ci(bootResults, type = "bca", index = 3)
```

```
## BOOTSTRAP CONFIDENCE INTERVAL CALCULATIONS
## Based on 2000 bootstrap replicates
## 
## CALL : 
## boot.ci(boot.out = bootResults, type = "bca", index = 3)
## 
## Intervals : 
## Level       BCa          
## 95%   (-0.7970,  0.7148 )  
## Calculations and Intervals on Original Scale
```

```
boot.ci(bootResults, type = "bca", index = 4)
```

```
## BOOTSTRAP CONFIDENCE INTERVAL CALCULATIONS
## Based on 2000 bootstrap replicates
## 
## CALL : 
## boot.ci(boot.out = bootResults, type = "bca", index = 4)
## 
## Intervals : 
## Level       BCa          
## 95%   (-0.8858,  0.4684 )  
## Calculations and Intervals on Original Scale
```

```
boot.ci(bootResults, type = "bca", index = 5)
```

```
## BOOTSTRAP CONFIDENCE INTERVAL CALCULATIONS
## Based on 2000 bootstrap replicates
## 
## CALL : 
## boot.ci(boot.out = bootResults, type = "bca", index = 5)
## 
## Intervals : 
## Level       BCa          
## 95%   (-0.358,  0.164 )  
## Calculations and Intervals on Original Scale
```

```
boot.ci(bootResults, type = "bca", index = 6)
```

```
## BOOTSTRAP CONFIDENCE INTERVAL CALCULATIONS
## Based on 2000 bootstrap replicates
## 
## CALL : 
## boot.ci(boot.out = bootResults, type = "bca", index = 6)
## 
## Intervals : 
## Level       BCa          
## 95%   (-0.0918,  1.5258 )  
## Calculations and Intervals on Original Scale
```

```
boot.ci(bootResults, type = "bca", index = 7)
```

```
## BOOTSTRAP CONFIDENCE INTERVAL CALCULATIONS
## Based on 2000 bootstrap replicates
## 
## CALL : 
## boot.ci(boot.out = bootResults, type = "bca", index = 7)
## 
## Intervals : 
## Level       BCa          
## 95%   ( 4.295,  5.875 )  
## Calculations and Intervals on Original Scale
```

```
round(confint(fit12), 2)
```

```
##             2.5 % 97.5 %
## (Intercept)  4.60   9.29
## Age         -0.09   0.11
## Gender      -0.76   0.71
## EDU         -0.89   0.49
## TECH_KN     -0.38   0.19
## CLASS1      -0.06   1.56
## CLASS2       4.30   5.92
```

```
# Compare the the control-only model and the regression model with the direct effect of design aids
anova(fit11, fit12)
```

```
## Analysis of Variance Table
## 
## Model 1: CORRECT_ALL ~ Age + Gender + EDU + TECH_KN
## Model 2: CORRECT_ALL ~ Age + Gender + EDU + TECH_KN + CLASS
##   Res.Df     RSS Df Sum of Sq      F    Pr(>F)    
## 1    185 1931.62                                  
## 2    183  971.74  2    959.88 90.383 < 2.2e-16 ***
## ---
## Signif. codes:  0 '***' 0.001 '**' 0.01 '*' 0.05 '.' 0.1 ' ' 1
```

```
# Hierarchical regression table
 stargazer(fit11, fit12, title = "Hierarchical Regression (H1)", type="text",
          star.char = c("+", "*", "**", "***"),
          star.cutoffs = c(.1, .05, .01, .001),
          notes= "+P<0.1; *P<0.05; **P<0.01; ***P<0.001", 
          notes.append = FALSE)
```

```
## 
## Hierarchical Regression (H1)
## ===============================================================
##                                 Dependent variable:            
##                     -------------------------------------------
##                                     CORRECT_ALL                
##                             (1)                   (2)          
## ---------------------------------------------------------------
## Age                        0.009                 0.012         
##                           (0.070)               (0.050)        
##                                                                
## Gender                    -0.325                -0.020         
##                           (0.521)               (0.372)        
##                                                                
## EDU                       -0.095                -0.203         
##                           (0.490)               (0.350)        
##                                                                
## TECH_KN                    0.065                -0.094         
##                           (0.204)               (0.146)        
##                                                                
## CLASS1                                          0.748+         
##                                                 (0.411)        
##                                                                
## CLASS2                                         5.111***        
##                                                 (0.410)        
##                                                                
## Constant                 8.741***              6.941***        
##                           (1.636)               (1.189)        
##                                                                
## ---------------------------------------------------------------
## Observations                190                   190          
## R2                         0.003                 0.499         
## Adjusted R2               -0.018                 0.482         
## Residual Std. Error  3.231 (df = 185)      2.304 (df = 183)    
## F Statistic         0.152 (df = 4; 185) 30.327*** (df = 6; 183)
## ===============================================================
## Note:                     +P<0.1; *P<0.05; **P<0.01; ***P<0.001
```

```
# Hierarchical regression table as word file
# stargazer(fit11, fit12, title = "Hierarchical Regression (H1)", type="html",
#          star.char = c("+", "*", "**", "***"),
#          star.cutoffs = c(.1, .05, .01, .001),
#          notes= "+P<0.1; *P<0.05; **P<0.01; ***P<0.001", 
#          out="Regression01.doc",
#          notes.append = FALSE,
#          single.row = T)

# Mean, Media, SD of selection accuracy for taxonomy, tags, and control group
Correct <- summaryBy(CORRECT_ALL ~ TREATMENT, data=Experiment, FUN=c(length, median, mean, sd))
Correct
```

```
##   TREATMENT CORRECT_ALL.length CORRECT_ALL.median CORRECT_ALL.mean
## 1   Control                 64                6.5         6.640625
## 2      Tags                 62                7.0         7.370968
## 3  Taxonomy                 64               12.0        11.718750
##   CORRECT_ALL.sd
## 1       2.445386
## 2       1.993710
## 3       2.380268
```

```
# Detailed analysisi of the different effect of decision aids on selection accuracy
# Test normal distribution of selection accuracy
shapiro.test(Experiment$CORRECT_ALL)
```

```
## 
##  Shapiro-Wilk normality test
## 
## data:  Experiment$CORRECT_ALL
## W = 0.9694, p-value = 0.000359
```

```
# Kruskal-Wallis test
kruskal.test(CORRECT_ALL ~ CLASS, data = Experiment)
```

```
## 
##  Kruskal-Wallis rank sum test
## 
## data:  CORRECT_ALL by CLASS
## Kruskal-Wallis chi-squared = 90.963, df = 2, p-value < 2.2e-16
```

```
# multile comparison test
# Mann-Whitney U test
pairwise.wilcox.test(Experiment$CORRECT_ALL, Experiment$CLASS, p.adjust.method="fdr")
```

```
## 
##  Pairwise comparisons using Wilcoxon rank sum test 
## 
## data:  Experiment$CORRECT_ALL and Experiment$CLASS 
## 
##   0       1      
## 1 0.079   -      
## 2 2.6e-16 1.8e-15
## 
## P value adjustment method: fdr
```

```
# Draw boxplots
# Selection accuracy as an dependent variable
MC <- Experiment %>% pairwise_wilcox_test(CORRECT_ALL ~ CLASS, p.adjust.method = "BH") 

p1 <- ggboxplot(Experiment, x = "CLASS", y = "CORRECT_ALL", color = "black", fill = "CLASS", palette="Blues") + 
  stat_pvalue_manual(MC, label = "p.adj", y.position = c(14, 16, 18)) +
  stat_compare_means(method = 'kruskal', label.y = 20) +
  theme_classic() +
  xlab("Decision aids") +
  scale_x_discrete(labels=c("No decision aid", "Tags-based", "Taxonomy-based")) +
  ylab("Selection accuracy") +
  theme(legend.position="null",
        axis.title.x = element_text(size=14),
        axis.text.x  = element_text(size=14),
        axis.title.y = element_text(size=14),
        axis.text.y  = element_text(size=14)) 
p1
```

```
# ggsave(filename = "p1.png", width = 6, height = 4, dpi = 300)
```

#### H2: Using a taxonomy-based decision aid leads to lower cognitive effort than using a tags-based decision aid, whereas using a tags-based decision aid leads to lower cognitive effort than using no decision aid at all.

```
# Hierarchical linear regression
# The regression of all control variables on cognitive effort
fit21 <- lm(COG_EFF ~ Age + Gender + EDU + TECH_KN, data = Experiment)
summary(fit21)
```

```
## 
## Call:
## lm(formula = COG_EFF ~ Age + Gender + EDU + TECH_KN, data = Experiment)
## 
## Residuals:
##     Min      1Q  Median      3Q     Max 
## -2.4793 -1.0716 -0.1629  1.1074  3.5330 
## 
## Coefficients:
##             Estimate Std. Error t value Pr(>|t|)    
## (Intercept)  2.84332    0.71560   3.973 0.000101 ***
## Age         -0.00208    0.03048  -0.068 0.945670    
## Gender       0.25349    0.22795   1.112 0.267554    
## EDU          0.11979    0.21421   0.559 0.576695    
## TECH_KN     -0.03959    0.08930  -0.443 0.658084    
## ---
## Signif. codes:  0 '***' 0.001 '**' 0.01 '*' 0.05 '.' 0.1 ' ' 1
## 
## Residual standard error: 1.413 on 185 degrees of freedom
## Multiple R-squared:  0.01116,    Adjusted R-squared:  -0.01022 
## F-statistic: 0.522 on 4 and 185 DF,  p-value: 0.7197
```

```
# The regression of the decision aids on cognitive effort
fit22 <- lm(COG_EFF ~ Age + Gender + EDU + TECH_KN + CLASS, data = Experiment)
summary(fit22)
```

```
## 
## Call:
## lm(formula = COG_EFF ~ Age + Gender + EDU + TECH_KN + CLASS, 
##     data = Experiment)
## 
## Residuals:
##     Min      1Q  Median      3Q     Max 
## -2.9265 -0.9479 -0.0939  0.7195  3.4846 
## 
## Coefficients:
##              Estimate Std. Error t value Pr(>|t|)    
## (Intercept)  3.654789   0.626160   5.837 2.38e-08 ***
## Age         -0.004052   0.026178  -0.155 0.877152    
## Gender       0.157074   0.196103   0.801 0.424185    
## EDU          0.164111   0.184034   0.892 0.373702    
## TECH_KN      0.014713   0.076967   0.191 0.848614    
## CLASS1      -0.805227   0.216509  -3.719 0.000266 ***
## CLASS2      -1.777175   0.215974  -8.229 3.45e-14 ***
## ---
## Signif. codes:  0 '***' 0.001 '**' 0.01 '*' 0.05 '.' 0.1 ' ' 1
## 
## Residual standard error: 1.213 on 183 degrees of freedom
## Multiple R-squared:  0.2788, Adjusted R-squared:  0.2551 
## F-statistic: 11.79 on 6 and 183 DF,  p-value: 3.672e-11
```

```
# Robust test of fit01

bootResults<-boot(statistic = bootReg, formula = CORRECT_ALL ~ Age + Gender + EDU + TECH_KN + CLASS, data = Experiment, R = 2000)
summary(bootResults)
```

```
## 
## Number of bootstrap replications R = 2000 
##    original   bootBias   bootSE   bootMed
## 1  6.941345 -0.0672336 1.119381  6.928212
## 2  0.011797  0.0031111 0.047806  0.012849
## 3 -0.020296 -0.0011173 0.393029 -0.011663
## 4 -0.203305 -0.0041285 0.330329 -0.216004
## 5 -0.093532  0.0023379 0.130480 -0.090873
## 6  0.747820 -0.0056162 0.404005  0.743866
## 7  5.110668 -0.0015365 0.433322  5.108076
```

```
# confidence intervals in the bootstrapped sample
boot.ci(bootResults, type = "bca", index = 1)
```

```
## BOOTSTRAP CONFIDENCE INTERVAL CALCULATIONS
## Based on 2000 bootstrap replicates
## 
## CALL : 
## boot.ci(boot.out = bootResults, type = "bca", index = 1)
## 
## Intervals : 
## Level       BCa          
## 95%   ( 4.587,  8.860 )  
## Calculations and Intervals on Original Scale
```

```
boot.ci(bootResults, type = "bca", index = 2)
```

```
## BOOTSTRAP CONFIDENCE INTERVAL CALCULATIONS
## Based on 2000 bootstrap replicates
## 
## CALL : 
## boot.ci(boot.out = bootResults, type = "bca", index = 2)
## 
## Intervals : 
## Level       BCa          
## 95%   (-0.0804,  0.1113 )  
## Calculations and Intervals on Original Scale
```

```
boot.ci(bootResults, type = "bca", index = 3)
```

```
## BOOTSTRAP CONFIDENCE INTERVAL CALCULATIONS
## Based on 2000 bootstrap replicates
## 
## CALL : 
## boot.ci(boot.out = bootResults, type = "bca", index = 3)
## 
## Intervals : 
## Level       BCa          
## 95%   (-0.8113,  0.7200 )  
## Calculations and Intervals on Original Scale
```

```
boot.ci(bootResults, type = "bca", index = 4)
```

```
## BOOTSTRAP CONFIDENCE INTERVAL CALCULATIONS
## Based on 2000 bootstrap replicates
## 
## CALL : 
## boot.ci(boot.out = bootResults, type = "bca", index = 4)
## 
## Intervals : 
## Level       BCa          
## 95%   (-0.7980,  0.5119 )  
## Calculations and Intervals on Original Scale
```

```
boot.ci(bootResults, type = "bca", index = 5)
```

```
## BOOTSTRAP CONFIDENCE INTERVAL CALCULATIONS
## Based on 2000 bootstrap replicates
## 
## CALL : 
## boot.ci(boot.out = bootResults, type = "bca", index = 5)
## 
## Intervals : 
## Level       BCa          
## 95%   (-0.3564,  0.1620 )  
## Calculations and Intervals on Original Scale
```

```
boot.ci(bootResults, type = "bca", index = 6)
```

```
## BOOTSTRAP CONFIDENCE INTERVAL CALCULATIONS
## Based on 2000 bootstrap replicates
## 
## CALL : 
## boot.ci(boot.out = bootResults, type = "bca", index = 6)
## 
## Intervals : 
## Level       BCa          
## 95%   (-0.0938,  1.5275 )  
## Calculations and Intervals on Original Scale
```

```
boot.ci(bootResults, type = "bca", index = 7)
```

```
## BOOTSTRAP CONFIDENCE INTERVAL CALCULATIONS
## Based on 2000 bootstrap replicates
## 
## CALL : 
## boot.ci(boot.out = bootResults, type = "bca", index = 7)
## 
## Intervals : 
## Level       BCa          
## 95%   ( 4.244,  5.985 )  
## Calculations and Intervals on Original Scale
```

```
round(confint(fit22), 2)
```

```
##             2.5 % 97.5 %
## (Intercept)  2.42   4.89
## Age         -0.06   0.05
## Gender      -0.23   0.54
## EDU         -0.20   0.53
## TECH_KN     -0.14   0.17
## CLASS1      -1.23  -0.38
## CLASS2      -2.20  -1.35
```

```
# Compare the control-only model and the regression model with the direct effect of decision aids
anova(fit21, fit22)
```

```
## Analysis of Variance Table
## 
## Model 1: COG_EFF ~ Age + Gender + EDU + TECH_KN
## Model 2: COG_EFF ~ Age + Gender + EDU + TECH_KN + CLASS
##   Res.Df    RSS Df Sum of Sq      F    Pr(>F)    
## 1    185 369.38                                  
## 2    183 269.42  2    99.961 33.949 2.885e-13 ***
## ---
## Signif. codes:  0 '***' 0.001 '**' 0.01 '*' 0.05 '.' 0.1 ' ' 1
```

```
# Hierarchical regression table
 stargazer(fit21, fit22, title = "Hierarchical Regression (H2)", type="text",
          star.char = c("+", "*", "**", "***"),
          star.cutoffs = c(.1, .05, .01, .001),
          notes= "+P<0.1; *P<0.05; **P<0.01; ***P<0.001", 
          notes.append = FALSE)
```

```
## 
## Hierarchical Regression (H2)
## ===============================================================
##                                 Dependent variable:            
##                     -------------------------------------------
##                                       COG_EFF                  
##                             (1)                   (2)          
## ---------------------------------------------------------------
## Age                       -0.002                -0.004         
##                           (0.030)               (0.026)        
##                                                                
## Gender                     0.253                 0.157         
##                           (0.228)               (0.196)        
##                                                                
## EDU                        0.120                 0.164         
##                           (0.214)               (0.184)        
##                                                                
## TECH_KN                   -0.040                 0.015         
##                           (0.089)               (0.077)        
##                                                                
## CLASS1                                         -0.805***       
##                                                 (0.217)        
##                                                                
## CLASS2                                         -1.777***       
##                                                 (0.216)        
##                                                                
## Constant                 2.843***              3.655***        
##                           (0.716)               (0.626)        
##                                                                
## ---------------------------------------------------------------
## Observations                190                   190          
## R2                         0.011                 0.279         
## Adjusted R2               -0.010                 0.255         
## Residual Std. Error  1.413 (df = 185)      1.213 (df = 183)    
## F Statistic         0.522 (df = 4; 185) 11.788*** (df = 6; 183)
## ===============================================================
## Note:                     +P<0.1; *P<0.05; **P<0.01; ***P<0.001
```

```
# Hierarchical regression table as word file
# stargazer(fit21, fit22, title = "Hierarchical Regression (H2)", type="html",
#          star.char = c("+", "*", "**", "***"),
#          star.cutoffs = c(.1, .05, .01, .001),
#          notes= "+P<0.1; *P<0.05; **P<0.01; ***P<0.001", 
#          out="Regression02.doc",
#          notes.append = FALSE,
#          single.row = T)

# Mean, Media, SD of cognitive effort for taxonomy, tags, and control group
CogEff <- summaryBy(COG_EFF ~ TREATMENT, data=Experiment, FUN=c(length, median, mean, sd))
CogEff
```

```
##   TREATMENT COG_EFF.length COG_EFF.median COG_EFF.mean COG_EFF.sd
## 1   Control             64            4.2     4.043750   1.171876
## 2      Tags             62            2.9     3.241935   1.294699
## 3  Taxonomy             64            2.0     2.265625   1.149771
```

```
# Detailed analysisi of the different effect of decision aids on cognitive effort
# Test normal distribution of cognitive effort
shapiro.test(Experiment$COG_EFF)
```

```
## 
##  Shapiro-Wilk normality test
## 
## data:  Experiment$COG_EFF
## W = 0.96326, p-value = 7.174e-05
```

```
# Kruskal-Wallis test
kruskal.test(COG_EFF ~ CLASS, data = Experiment)
```

```
## 
##  Kruskal-Wallis rank sum test
## 
## data:  COG_EFF by CLASS
## Kruskal-Wallis chi-squared = 53.105, df = 2, p-value = 2.941e-12
```

```
# multile comparison test
# Mann-Whitney U test
pairwise.wilcox.test(Experiment$COG_EFF, Experiment$CLASS, p.adjust.method="fdr")
```

```
## 
##  Pairwise comparisons using Wilcoxon rank sum test 
## 
## data:  Experiment$COG_EFF and Experiment$CLASS 
## 
##   0       1      
## 1 0.00035 -      
## 2 1.6e-11 1.9e-05
## 
## P value adjustment method: fdr
```

```
# Draw boxplots
# Selection accuracy as an dependent variable
MD <- Experiment %>% pairwise_wilcox_test(COG_EFF ~ CLASS, p.adjust.method = "BH") 

p2 <- ggboxplot(Experiment, x = "CLASS", y = "COG_EFF", color = "black", fill = "CLASS", palette="Blues") + 
  stat_pvalue_manual(MD, label = "p.adj", y.position = c(9, 8, 7)) +
  stat_compare_means(method = 'kruskal', label.y = 10) +
  theme_classic() +
  xlab("Decision aids") +
  scale_x_discrete(labels=c("No decision aid", "Tags-based", "Taxonomy-based")) +
  ylab("Cognitive effort") +
  theme(legend.position="null",
        axis.title.x = element_text(size=14),
        axis.text.x  = element_text(size=14),
        axis.title.y = element_text(size=14),
        axis.text.y  = element_text(size=14)) 
p2
```

```
# ggsave(filename = "p2.png", width = 6, height = 4, dpi = 300)

# ggarrange(p1,p2)
# ggsave(filename = "p12.png", width = 11, height = 5, dpi = 300)
```

#### H3a: An increase in cognitive effort leads to lower selection accuracy.

```
# The regression of cognitive effort on selection accuracy
fit31 <- lm(CORRECT_ALL ~ Age + Gender + EDU + TECH_KN + COG_EFF, data = Experiment)
summary(fit31)
```

```
## 
## Call:
## lm(formula = CORRECT_ALL ~ Age + Gender + EDU + TECH_KN + COG_EFF, 
##     data = Experiment)
## 
## Residuals:
##    Min     1Q Median     3Q    Max 
## -8.036 -1.913  0.133  1.988  6.934 
## 
## Coefficients:
##             Estimate Std. Error t value Pr(>|t|)    
## (Intercept) 11.75699    1.51440   7.763 5.56e-13 ***
## Age          0.00727    0.06192   0.117    0.907    
## Gender      -0.05563    0.46459  -0.120    0.905    
## EDU          0.03211    0.43550   0.074    0.941    
## TECH_KN      0.02331    0.18151   0.128    0.898    
## COG_EFF     -1.06078    0.14935  -7.103 2.59e-11 ***
## ---
## Signif. codes:  0 '***' 0.001 '**' 0.01 '*' 0.05 '.' 0.1 ' ' 1
## 
## Residual standard error: 2.87 on 184 degrees of freedom
## Multiple R-squared:  0.2178, Adjusted R-squared:  0.1965 
## F-statistic: 10.24 on 5 and 184 DF,  p-value: 1.123e-08
```

```
# The regression of decision aids and cognitive effort on selection accuracy
fit32 <- lm(CORRECT_ALL ~ Age + Gender + EDU + TECH_KN + CLASS + COG_EFF, data = Experiment)
summary(fit32)
```

```
## 
## Call:
## lm(formula = CORRECT_ALL ~ Age + Gender + EDU + TECH_KN + CLASS + 
##     COG_EFF, data = Experiment)
## 
## Residuals:
##     Min      1Q  Median      3Q     Max 
## -5.5892 -1.3473 -0.0079  1.6387  4.7114 
## 
## Coefficients:
##             Estimate Std. Error t value Pr(>|t|)    
## (Intercept)  8.28491    1.27415   6.502 7.39e-10 ***
## Age          0.01031    0.04891   0.211  0.83334    
## Gender       0.03745    0.36703   0.102  0.91885    
## EDU         -0.14298    0.34459  -0.415  0.67869    
## TECH_KN     -0.08812    0.14382  -0.613  0.54081    
## CLASS1       0.45180    0.41953   1.077  0.28293    
## CLASS2       4.45735    0.47231   9.437  < 2e-16 ***
## COG_EFF     -0.36762    0.13811  -2.662  0.00847 ** 
## ---
## Signif. codes:  0 '***' 0.001 '**' 0.01 '*' 0.05 '.' 0.1 ' ' 1
## 
## Residual standard error: 2.267 on 182 degrees of freedom
## Multiple R-squared:  0.5174, Adjusted R-squared:  0.4988 
## F-statistic: 27.87 on 7 and 182 DF,  p-value: < 2.2e-16
```

```
# Robust test of fit31
bootResults<-boot(statistic = bootReg, formula = CORRECT_ALL ~ Age + Gender + EDU + TECH_KN + COG_EFF, data = Experiment, R = 2000)
summary(bootResults)
```

```
## 
## Number of bootstrap replications R = 2000 
##     original   bootBias  bootSE    bootMed
## 1 11.7569907 -0.0883815 1.46881 11.7355094
## 2  0.0072703  0.0024812 0.05228  0.0064851
## 3 -0.0556299  0.0221721 0.46163 -0.0315088
## 4  0.0321148 -0.0073398 0.40881  0.0142291
## 5  0.0233134  0.0051116 0.17735  0.0264901
## 6 -1.0607777  0.0002026 0.14574 -1.0601496
```

```
# confidence intervals in the bootstrapped sample
boot.ci(bootResults, type = "bca", index = 1)
```

```
## BOOTSTRAP CONFIDENCE INTERVAL CALCULATIONS
## Based on 2000 bootstrap replicates
## 
## CALL : 
## boot.ci(boot.out = bootResults, type = "bca", index = 1)
## 
## Intervals : 
## Level       BCa          
## 95%   ( 8.59, 14.34 )  
## Calculations and Intervals on Original Scale
```

```
boot.ci(bootResults, type = "bca", index = 2)
```

```
## BOOTSTRAP CONFIDENCE INTERVAL CALCULATIONS
## Based on 2000 bootstrap replicates
## 
## CALL : 
## boot.ci(boot.out = bootResults, type = "bca", index = 2)
## 
## Intervals : 
## Level       BCa          
## 95%   (-0.0833,  0.1245 )  
## Calculations and Intervals on Original Scale
```

```
boot.ci(bootResults, type = "bca", index = 3)
```

```
## BOOTSTRAP CONFIDENCE INTERVAL CALCULATIONS
## Based on 2000 bootstrap replicates
## 
## CALL : 
## boot.ci(boot.out = bootResults, type = "bca", index = 3)
## 
## Intervals : 
## Level       BCa          
## 95%   (-1.0056,  0.8210 )  
## Calculations and Intervals on Original Scale
```

```
boot.ci(bootResults, type = "bca", index = 4)
```

```
## BOOTSTRAP CONFIDENCE INTERVAL CALCULATIONS
## Based on 2000 bootstrap replicates
## 
## CALL : 
## boot.ci(boot.out = bootResults, type = "bca", index = 4)
## 
## Intervals : 
## Level       BCa          
## 95%   (-0.7276,  0.8760 )  
## Calculations and Intervals on Original Scale
```

```
boot.ci(bootResults, type = "bca", index = 5)
```

```
## BOOTSTRAP CONFIDENCE INTERVAL CALCULATIONS
## Based on 2000 bootstrap replicates
## 
## CALL : 
## boot.ci(boot.out = bootResults, type = "bca", index = 5)
## 
## Intervals : 
## Level       BCa          
## 95%   (-0.3248,  0.3816 )  
## Calculations and Intervals on Original Scale
```

```
boot.ci(bootResults, type = "bca", index = 6)
```

```
## BOOTSTRAP CONFIDENCE INTERVAL CALCULATIONS
## Based on 2000 bootstrap replicates
## 
## CALL : 
## boot.ci(boot.out = bootResults, type = "bca", index = 6)
## 
## Intervals : 
## Level       BCa          
## 95%   (-1.338, -0.777 )  
## Calculations and Intervals on Original Scale
```

```
round(confint(fit31), 2)
```

```
##             2.5 % 97.5 %
## (Intercept)  8.77  14.74
## Age         -0.11   0.13
## Gender      -0.97   0.86
## EDU         -0.83   0.89
## TECH_KN     -0.33   0.38
## COG_EFF     -1.36  -0.77
```

```
# Robust test of fit32
bootResults<-boot(statistic = bootReg, formula = CORRECT_ALL ~ Age + Gender + EDU + TECH_KN + CLASS + COG_EFF, data = Experiment, R = 2000)
summary(bootResults)
```

```
## 
## Number of bootstrap replications R = 2000 
##    original    bootBias   bootSE   bootMed
## 1  8.284912 -0.04220902 1.288171  8.267443
## 2  0.010307  0.00471688 0.045736  0.013175
## 3  0.037448 -0.02617044 0.394727  0.016634
## 4 -0.142975 -0.00961727 0.319155 -0.157392
## 5 -0.088124 -0.00031779 0.131929 -0.083817
## 6  0.451804 -0.00578648 0.391580  0.455420
## 7  4.457346 -0.00952408 0.510537  4.456541
## 8 -0.367618 -0.00459269 0.153958 -0.376935
```

```
# confidence intervals in the bootstrapped sample
boot.ci(bootResults, type = "bca", index = 1)
```

```
## BOOTSTRAP CONFIDENCE INTERVAL CALCULATIONS
## Based on 2000 bootstrap replicates
## 
## CALL : 
## boot.ci(boot.out = bootResults, type = "bca", index = 1)
## 
## Intervals : 
## Level       BCa          
## 95%   ( 5.622, 10.650 )  
## Calculations and Intervals on Original Scale
```

```
boot.ci(bootResults, type = "bca", index = 2)
```

```
## BOOTSTRAP CONFIDENCE INTERVAL CALCULATIONS
## Based on 2000 bootstrap replicates
## 
## CALL : 
## boot.ci(boot.out = bootResults, type = "bca", index = 2)
## 
## Intervals : 
## Level       BCa          
## 95%   (-0.0780,  0.1004 )  
## Calculations and Intervals on Original Scale
```

```
boot.ci(bootResults, type = "bca", index = 3)
```

```
## BOOTSTRAP CONFIDENCE INTERVAL CALCULATIONS
## Based on 2000 bootstrap replicates
## 
## CALL : 
## boot.ci(boot.out = bootResults, type = "bca", index = 3)
## 
## Intervals : 
## Level       BCa          
## 95%   (-0.7528,  0.7875 )  
## Calculations and Intervals on Original Scale
```

```
boot.ci(bootResults, type = "bca", index = 4)
```

```
## BOOTSTRAP CONFIDENCE INTERVAL CALCULATIONS
## Based on 2000 bootstrap replicates
## 
## CALL : 
## boot.ci(boot.out = bootResults, type = "bca", index = 4)
## 
## Intervals : 
## Level       BCa          
## 95%   (-0.7355,  0.5257 )  
## Calculations and Intervals on Original Scale
```

```
boot.ci(bootResults, type = "bca", index = 5)
```

```
## BOOTSTRAP CONFIDENCE INTERVAL CALCULATIONS
## Based on 2000 bootstrap replicates
## 
## CALL : 
## boot.ci(boot.out = bootResults, type = "bca", index = 5)
## 
## Intervals : 
## Level       BCa          
## 95%   (-0.3621,  0.1547 )  
## Calculations and Intervals on Original Scale
```

```
boot.ci(bootResults, type = "bca", index = 6)
```

```
## BOOTSTRAP CONFIDENCE INTERVAL CALCULATIONS
## Based on 2000 bootstrap replicates
## 
## CALL : 
## boot.ci(boot.out = bootResults, type = "bca", index = 6)
## 
## Intervals : 
## Level       BCa          
## 95%   (-0.3149,  1.2060 )  
## Calculations and Intervals on Original Scale
```

```
boot.ci(bootResults, type = "bca", index = 7)
```

```
## BOOTSTRAP CONFIDENCE INTERVAL CALCULATIONS
## Based on 2000 bootstrap replicates
## 
## CALL : 
## boot.ci(boot.out = bootResults, type = "bca", index = 7)
## 
## Intervals : 
## Level       BCa          
## 95%   ( 3.424,  5.437 )  
## Calculations and Intervals on Original Scale
```

```
boot.ci(bootResults, type = "bca", index = 8)
```

```
## BOOTSTRAP CONFIDENCE INTERVAL CALCULATIONS
## Based on 2000 bootstrap replicates
## 
## CALL : 
## boot.ci(boot.out = bootResults, type = "bca", index = 8)
## 
## Intervals : 
## Level       BCa          
## 95%   (-0.6662, -0.0558 )  
## Calculations and Intervals on Original Scale
```

```
round(confint(fit32), 2)
```

```
##             2.5 % 97.5 %
## (Intercept)  5.77  10.80
## Age         -0.09   0.11
## Gender      -0.69   0.76
## EDU         -0.82   0.54
## TECH_KN     -0.37   0.20
## CLASS1      -0.38   1.28
## CLASS2       3.53   5.39
## COG_EFF     -0.64  -0.10
```

```
# Compare the control-only model and the regression model with the direct effect of cognitive effort
anova(fit11, fit31)
```

```
## Analysis of Variance Table
## 
## Model 1: CORRECT_ALL ~ Age + Gender + EDU + TECH_KN
## Model 2: CORRECT_ALL ~ Age + Gender + EDU + TECH_KN + COG_EFF
##   Res.Df    RSS Df Sum of Sq      F    Pr(>F)    
## 1    185 1931.6                                  
## 2    184 1516.0  1    415.64 50.448 2.591e-11 ***
## ---
## Signif. codes:  0 '***' 0.001 '**' 0.01 '*' 0.05 '.' 0.1 ' ' 1
```

#### H3b: Cognitive effort mediates the effect of decision aids toward selection accuracy.

```
# Effect of decision aids on cognitive effort
fitM <- lm(COG_EFF ~ Age + Gender + EDU + TECH_KN + CLASS, data = Experiment)
summary(fitM)
```

```
## 
## Call:
## lm(formula = COG_EFF ~ Age + Gender + EDU + TECH_KN + CLASS, 
##     data = Experiment)
## 
## Residuals:
##     Min      1Q  Median      3Q     Max 
## -2.9265 -0.9479 -0.0939  0.7195  3.4846 
## 
## Coefficients:
##              Estimate Std. Error t value Pr(>|t|)    
## (Intercept)  3.654789   0.626160   5.837 2.38e-08 ***
## Age         -0.004052   0.026178  -0.155 0.877152    
## Gender       0.157074   0.196103   0.801 0.424185    
## EDU          0.164111   0.184034   0.892 0.373702    
## TECH_KN      0.014713   0.076967   0.191 0.848614    
## CLASS1      -0.805227   0.216509  -3.719 0.000266 ***
## CLASS2      -1.777175   0.215974  -8.229 3.45e-14 ***
## ---
## Signif. codes:  0 '***' 0.001 '**' 0.01 '*' 0.05 '.' 0.1 ' ' 1
## 
## Residual standard error: 1.213 on 183 degrees of freedom
## Multiple R-squared:  0.2788, Adjusted R-squared:  0.2551 
## F-statistic: 11.79 on 6 and 183 DF,  p-value: 3.672e-11
```

```
# Effect of decision aids and cognitive effort on selection accuracy
fitY <- lm(CORRECT_ALL ~ Age + Gender + EDU + TECH_KN + CLASS + COG_EFF, data = Experiment)
summary(fitY)
```

```
## 
## Call:
## lm(formula = CORRECT_ALL ~ Age + Gender + EDU + TECH_KN + CLASS + 
##     COG_EFF, data = Experiment)
## 
## Residuals:
##     Min      1Q  Median      3Q     Max 
## -5.5892 -1.3473 -0.0079  1.6387  4.7114 
## 
## Coefficients:
##             Estimate Std. Error t value Pr(>|t|)    
## (Intercept)  8.28491    1.27415   6.502 7.39e-10 ***
## Age          0.01031    0.04891   0.211  0.83334    
## Gender       0.03745    0.36703   0.102  0.91885    
## EDU         -0.14298    0.34459  -0.415  0.67869    
## TECH_KN     -0.08812    0.14382  -0.613  0.54081    
## CLASS1       0.45180    0.41953   1.077  0.28293    
## CLASS2       4.45735    0.47231   9.437  < 2e-16 ***
## COG_EFF     -0.36762    0.13811  -2.662  0.00847 ** 
## ---
## Signif. codes:  0 '***' 0.001 '**' 0.01 '*' 0.05 '.' 0.1 ' ' 1
## 
## Residual standard error: 2.267 on 182 degrees of freedom
## Multiple R-squared:  0.5174, Adjusted R-squared:  0.4988 
## F-statistic: 27.87 on 7 and 182 DF,  p-value: < 2.2e-16
```

```
# Test the mediating effect of cognitive effort between tags and selection accuracy
fitMedBoot1 <- mediate(fitM, fitY, boot = TRUE, treat = "CLASS", mediator = "COG_EFF", control.value = 0, treat.value = 1)
```

```
## Running nonparametric bootstrap
```

```
summary(fitMedBoot1)
```

```
## 
## Causal Mediation Analysis 
## 
## Nonparametric Bootstrap Confidence Intervals with the Percentile Method
## 
##                Estimate 95% CI Lower 95% CI Upper p-value  
## ACME             0.2960       0.0444         0.63   0.018 *
## ADE              0.4518      -0.3953         1.24   0.266  
## Total Effect     0.7478      -0.0657         1.53   0.070 .
## Prop. Mediated   0.3958      -1.1033         2.02   0.088 .
## ---
## Signif. codes:  0 '***' 0.001 '**' 0.01 '*' 0.05 '.' 0.1 ' ' 1
## 
## Sample Size Used: 190 
## 
## 
## Simulations: 1000
```

```
# Test the mediating effect of cognitive effort between taxonomy and selection accuracy
fitMedBoot2 <- mediate(fitM, fitY, boot = TRUE, treat = "CLASS", mediator = "COG_EFF", control.value = 0, treat.value = 2)
```

```
## Running nonparametric bootstrap
```

```
summary(fitMedBoot2)
```

```
## 
## Causal Mediation Analysis 
## 
## Nonparametric Bootstrap Confidence Intervals with the Percentile Method
## 
##                Estimate 95% CI Lower 95% CI Upper p-value    
## ACME             0.6533       0.0600         1.24   0.026 *  
## ADE              4.4573       3.5002         5.51  <2e-16 ***
## Total Effect     5.1107       4.3127         6.00  <2e-16 ***
## Prop. Mediated   0.1278       0.0108         0.25   0.026 *  
## ---
## Signif. codes:  0 '***' 0.001 '**' 0.01 '*' 0.05 '.' 0.1 ' ' 1
## 
## Sample Size Used: 190 
## 
## 
## Simulations: 1000
```

```
# Hierarchical regression table
 stargazer(fit11, fit31, fit32, title = "Hierarchical Regression (H3b)", type="text",
          star.char = c("+", "*", "**", "***"),
          star.cutoffs = c(.1, .05, .01, .001),
          notes= "+P<0.1; *P<0.05; **P<0.01; ***P<0.001", 
          notes.append = FALSE)
```

```
## 
## Hierarchical Regression (H3b)
## =======================================================================================
##                                             Dependent variable:                        
##                     -------------------------------------------------------------------
##                                                 CORRECT_ALL                            
##                             (1)                   (2)                     (3)          
## ---------------------------------------------------------------------------------------
## Age                        0.009                 0.007                   0.010         
##                           (0.070)               (0.062)                 (0.049)        
##                                                                                        
## Gender                    -0.325                -0.056                   0.037         
##                           (0.521)               (0.465)                 (0.367)        
##                                                                                        
## EDU                       -0.095                 0.032                  -0.143         
##                           (0.490)               (0.436)                 (0.345)        
##                                                                                        
## TECH_KN                    0.065                 0.023                  -0.088         
##                           (0.204)               (0.182)                 (0.144)        
##                                                                                        
## CLASS1                                                                   0.452         
##                                                                         (0.420)        
##                                                                                        
## CLASS2                                                                 4.457***        
##                                                                         (0.472)        
##                                                                                        
## COG_EFF                                        -1.061***               -0.368**        
##                                                 (0.149)                 (0.138)        
##                                                                                        
## Constant                 8.741***              11.757***               8.285***        
##                           (1.636)               (1.514)                 (1.274)        
##                                                                                        
## ---------------------------------------------------------------------------------------
## Observations                190                   190                     190          
## R2                         0.003                 0.218                   0.517         
## Adjusted R2               -0.018                 0.196                   0.499         
## Residual Std. Error  3.231 (df = 185)      2.870 (df = 184)        2.267 (df = 182)    
## F Statistic         0.152 (df = 4; 185) 10.244*** (df = 5; 184) 27.871*** (df = 7; 182)
## =======================================================================================
## Note:                                             +P<0.1; *P<0.05; **P<0.01; ***P<0.001
```

```
# Hierarchical regression table as word file
# stargazer(fit11, fit31, fit32, title = "Hierarchical Regression (H3a, H3b)", type="html",
#          star.char = c("+", "*", "**", "***"),
#          star.cutoffs = c(.1, .05, .01, .001),
#          notes= "+P<0.1; *P<0.05; **P<0.01; ***P<0.001", 
#          out="Regression03.doc",
#          notes.append = FALSE,
#          single.row = T)
```

#### H4a: The positive effect of using a tags-based decision aid on selection accuracy is moderated by an individual’s intuitive decision style, whereby the effect is stronger for individuals with a high intuitive decision style.

#### H4b: The positive effect of using a taxonomy-based decision aid on selection accuracy is moderated by an individual’s rational decision style, whereby the effect is stronger for individuals with a high rational decision style.

```
# Test whether there was no difference in the rational and intuitive decision-making styles across the three experiment groups 

kruskal.test(DES_ST_RA ~ CLASS, data = Experiment)
```

```
## 
##  Kruskal-Wallis rank sum test
## 
## data:  DES_ST_RA by CLASS
## Kruskal-Wallis chi-squared = 0.95234, df = 2, p-value = 0.6212
```

```
pairwise.wilcox.test(Experiment$DES_ST_RA, Experiment$CLASS, p.adjust.method="fdr")
```

```
## 
##  Pairwise comparisons using Wilcoxon rank sum test 
## 
## data:  Experiment$DES_ST_RA and Experiment$CLASS 
## 
##   0    1   
## 1 0.99 -   
## 2 0.62 0.62
## 
## P value adjustment method: fdr
```

```
kruskal.test(DES_ST_IN ~ CLASS, data = Experiment)
```

```
## 
##  Kruskal-Wallis rank sum test
## 
## data:  DES_ST_IN by CLASS
## Kruskal-Wallis chi-squared = 1.3939, df = 2, p-value = 0.4981
```

```
pairwise.wilcox.test(Experiment$DES_ST_IN, Experiment$CLASS, p.adjust.method="fdr")
```

```
## 
##  Pairwise comparisons using Wilcoxon rank sum test 
## 
## data:  Experiment$DES_ST_IN and Experiment$CLASS 
## 
##   0    1   
## 1 0.85 -   
## 2 0.56 0.56
## 
## P value adjustment method: fdr
```

```
# Regression of the moderating effect of intuitive decision style
fit41 <- lm(CORRECT_ALL ~ Age + Gender + EDU + TECH_KN + CLASS * DES_ST_IN, data = Experiment)
summary(fit41)
```

```
## 
## Call:
## lm(formula = CORRECT_ALL ~ Age + Gender + EDU + TECH_KN + CLASS * 
##     DES_ST_IN, data = Experiment)
## 
## Residuals:
##    Min     1Q Median     3Q    Max 
## -6.666 -1.446  0.232  1.820  4.587 
## 
## Coefficients:
##                   Estimate Std. Error t value Pr(>|t|)    
## (Intercept)       7.490606   1.431341   5.233  4.6e-07 ***
## Age               0.009827   0.050060   0.196 0.844586    
## Gender            0.059456   0.380318   0.156 0.875947    
## EDU              -0.206378   0.351674  -0.587 0.558044    
## TECH_KN          -0.065755   0.148733  -0.442 0.658949    
## CLASS1            0.909820   1.326485   0.686 0.493666    
## CLASS2            4.784621   1.331068   3.595 0.000419 ***
## DES_ST_IN        -0.206742   0.259049  -0.798 0.425875    
## CLASS1:DES_ST_IN -0.051416   0.383870  -0.134 0.893598    
## CLASS2:DES_ST_IN  0.104015   0.374606   0.278 0.781588    
## ---
## Signif. codes:  0 '***' 0.001 '**' 0.01 '*' 0.05 '.' 0.1 ' ' 1
## 
## Residual standard error: 2.314 on 180 degrees of freedom
## Multiple R-squared:  0.5028, Adjusted R-squared:  0.478 
## F-statistic: 20.23 on 9 and 180 DF,  p-value: < 2.2e-16
```

```
# Robust test of fit41
bootResults<-boot(statistic = bootReg, formula = CORRECT_ALL ~ Age + Gender + EDU + TECH_KN + CLASS * DES_ST_IN, data = Experiment, R = 2000)
summary(bootResults)
```

```
## 
## Number of bootstrap replications R = 2000 
##      original   bootBias   bootSE   bootMed
## 1   7.4906056 -0.1520899 1.447595  7.356094
## 2   0.0098274  0.0044454 0.046299  0.012122
## 3   0.0594558  0.0036702 0.389502  0.060316
## 4  -0.2063778 -0.0013884 0.343495 -0.208478
## 5  -0.0657546  0.0083807 0.138295 -0.053825
## 6   0.9098205  0.0718998 1.285382  0.977838
## 7   4.7846207  0.0582261 1.446641  4.820567
## 8  -0.2067419  0.0051906 0.300899 -0.196575
## 9  -0.0514164 -0.0209764 0.374864 -0.075044
## 10  0.1040153 -0.0150619 0.432114  0.096557
```

```
# confidence intervals in the bootstrapped sample
boot.ci(bootResults, type = "bca", index = 1)
```

```
## BOOTSTRAP CONFIDENCE INTERVAL CALCULATIONS
## Based on 2000 bootstrap replicates
## 
## CALL : 
## boot.ci(boot.out = bootResults, type = "bca", index = 1)
## 
## Intervals : 
## Level       BCa          
## 95%   ( 4.786, 10.412 )  
## Calculations and Intervals on Original Scale
```

```
boot.ci(bootResults, type = "bca", index = 2)
```

```
## BOOTSTRAP CONFIDENCE INTERVAL CALCULATIONS
## Based on 2000 bootstrap replicates
## 
## CALL : 
## boot.ci(boot.out = bootResults, type = "bca", index = 2)
## 
## Intervals : 
## Level       BCa          
## 95%   (-0.0800,  0.1032 )  
## Calculations and Intervals on Original Scale
```

```
boot.ci(bootResults, type = "bca", index = 3)
```

```
## BOOTSTRAP CONFIDENCE INTERVAL CALCULATIONS
## Based on 2000 bootstrap replicates
## 
## CALL : 
## boot.ci(boot.out = bootResults, type = "bca", index = 3)
## 
## Intervals : 
## Level       BCa          
## 95%   (-0.7022,  0.8193 )  
## Calculations and Intervals on Original Scale
```

```
boot.ci(bootResults, type = "bca", index = 4)
```

```
## BOOTSTRAP CONFIDENCE INTERVAL CALCULATIONS
## Based on 2000 bootstrap replicates
## 
## CALL : 
## boot.ci(boot.out = bootResults, type = "bca", index = 4)
## 
## Intervals : 
## Level       BCa          
## 95%   (-0.8854,  0.4813 )  
## Calculations and Intervals on Original Scale
```

```
boot.ci(bootResults, type = "bca", index = 5)
```

```
## BOOTSTRAP CONFIDENCE INTERVAL CALCULATIONS
## Based on 2000 bootstrap replicates
## 
## CALL : 
## boot.ci(boot.out = bootResults, type = "bca", index = 5)
## 
## Intervals : 
## Level       BCa          
## 95%   (-0.3516,  0.1865 )  
## Calculations and Intervals on Original Scale
```

```
boot.ci(bootResults, type = "bca", index = 6)
```

```
## BOOTSTRAP CONFIDENCE INTERVAL CALCULATIONS
## Based on 2000 bootstrap replicates
## 
## CALL : 
## boot.ci(boot.out = bootResults, type = "bca", index = 6)
## 
## Intervals : 
## Level       BCa          
## 95%   (-1.7344,  3.2934 )  
## Calculations and Intervals on Original Scale
```

```
boot.ci(bootResults, type = "bca", index = 7)
```

```
## BOOTSTRAP CONFIDENCE INTERVAL CALCULATIONS
## Based on 2000 bootstrap replicates
## 
## CALL : 
## boot.ci(boot.out = bootResults, type = "bca", index = 7)
## 
## Intervals : 
## Level       BCa          
## 95%   ( 1.876,  7.697 )  
## Calculations and Intervals on Original Scale
```

```
boot.ci(bootResults, type = "bca", index = 8)
```

```
## BOOTSTRAP CONFIDENCE INTERVAL CALCULATIONS
## Based on 2000 bootstrap replicates
## 
## CALL : 
## boot.ci(boot.out = bootResults, type = "bca", index = 8)
## 
## Intervals : 
## Level       BCa          
## 95%   (-0.8234,  0.3426 )  
## Calculations and Intervals on Original Scale
```

```
boot.ci(bootResults, type = "bca", index = 9)
```

```
## BOOTSTRAP CONFIDENCE INTERVAL CALCULATIONS
## Based on 2000 bootstrap replicates
## 
## CALL : 
## boot.ci(boot.out = bootResults, type = "bca", index = 9)
## 
## Intervals : 
## Level       BCa          
## 95%   (-0.7198,  0.7084 )  
## Calculations and Intervals on Original Scale
```

```
boot.ci(bootResults, type = "bca", index = 10)
```

```
## BOOTSTRAP CONFIDENCE INTERVAL CALCULATIONS
## Based on 2000 bootstrap replicates
## 
## CALL : 
## boot.ci(boot.out = bootResults, type = "bca", index = 10)
## 
## Intervals : 
## Level       BCa          
## 95%   (-0.7616,  0.9746 )  
## Calculations and Intervals on Original Scale
```

```
# confidence intervals in fit41
round(confint(fit41), 2)
```

```
##                  2.5 % 97.5 %
## (Intercept)       4.67  10.31
## Age              -0.09   0.11
## Gender           -0.69   0.81
## EDU              -0.90   0.49
## TECH_KN          -0.36   0.23
## CLASS1           -1.71   3.53
## CLASS2            2.16   7.41
## DES_ST_IN        -0.72   0.30
## CLASS1:DES_ST_IN -0.81   0.71
## CLASS2:DES_ST_IN -0.64   0.84
```

```
# Compare the control-only model and the regression model with intuitive decision style as a moderator
anova(fit12, fit41)
```

```
## Analysis of Variance Table
## 
## Model 1: CORRECT_ALL ~ Age + Gender + EDU + TECH_KN + CLASS
## Model 2: CORRECT_ALL ~ Age + Gender + EDU + TECH_KN + CLASS * DES_ST_IN
##   Res.Df    RSS Df Sum of Sq      F Pr(>F)
## 1    183 971.74                           
## 2    180 963.49  3    8.2474 0.5136 0.6734
```

```
# Regression of the moderating effect of rational decision style
fit42 <- lm(CORRECT_ALL ~ Age + Gender + EDU + TECH_KN + CLASS * DES_ST_RA, data = Experiment)
summary(fit42)
```

```
## 
## Call:
## lm(formula = CORRECT_ALL ~ Age + Gender + EDU + TECH_KN + CLASS * 
##     DES_ST_RA, data = Experiment)
## 
## Residuals:
##     Min      1Q  Median      3Q     Max 
## -6.6615 -1.4566  0.2734  1.6063  4.5195 
## 
## Coefficients:
##                  Estimate Std. Error t value Pr(>|t|)    
## (Intercept)       3.40143    1.93251   1.760 0.080087 .  
## Age               0.02640    0.04973   0.531 0.596165    
## Gender           -0.03317    0.36923  -0.090 0.928512    
## EDU              -0.29551    0.34738  -0.851 0.396072    
## TECH_KN          -0.09058    0.14532  -0.623 0.533845    
## CLASS1            1.41530    2.59383   0.546 0.585987    
## CLASS2            9.39743    2.41665   3.889 0.000142 ***
## DES_ST_RA         0.62390    0.27180   2.295 0.022862 *  
## CLASS1:DES_ST_RA -0.13340    0.47169  -0.283 0.777651    
## CLASS2:DES_ST_RA -0.79261    0.43351  -1.828 0.069149 .  
## ---
## Signif. codes:  0 '***' 0.001 '**' 0.01 '*' 0.05 '.' 0.1 ' ' 1
## 
## Residual standard error: 2.279 on 180 degrees of freedom
## Multiple R-squared:  0.5177, Adjusted R-squared:  0.4936 
## F-statistic: 21.47 on 9 and 180 DF,  p-value: < 2.2e-16
```

```
# Robust test of fit42
bootResults<-boot(statistic = bootReg, formula = CORRECT_ALL ~ Age + Gender + EDU + TECH_KN + CLASS * DES_ST_RA, data = Experiment, R = 2000)
summary(bootResults)
```

```
## 
## Number of bootstrap replications R = 2000 
##     original   bootBias   bootSE   bootMed
## 1   3.401431 -0.1221850 1.712896  3.391023
## 2   0.026399  0.0041516 0.044599  0.028375
## 3  -0.033173 -0.0041415 0.394373 -0.023675
## 4  -0.295513 -0.0210370 0.337752 -0.322111
## 5  -0.090585  0.0027023 0.134240 -0.089121
## 6   1.415304 -0.0237747 2.504216  1.387778
## 7   9.397433  0.1114361 2.398781  9.461709
## 8   0.623902  0.0081275 0.247180  0.616561
## 9  -0.133396  0.0063748 0.453412 -0.128804
## 10 -0.792612 -0.0173807 0.425534 -0.809280
```

```
# confidence intervals in the bootstrapped sample
boot.ci(bootResults, type = "bca", index = 1)
```

```
## BOOTSTRAP CONFIDENCE INTERVAL CALCULATIONS
## Based on 2000 bootstrap replicates
## 
## CALL : 
## boot.ci(boot.out = bootResults, type = "bca", index = 1)
## 
## Intervals : 
## Level       BCa          
## 95%   (-0.159,  6.549 )  
## Calculations and Intervals on Original Scale
```

```
boot.ci(bootResults, type = "bca", index = 2)
```

```
## BOOTSTRAP CONFIDENCE INTERVAL CALCULATIONS
## Based on 2000 bootstrap replicates
## 
## CALL : 
## boot.ci(boot.out = bootResults, type = "bca", index = 2)
## 
## Intervals : 
## Level       BCa          
## 95%   (-0.0582,  0.1153 )  
## Calculations and Intervals on Original Scale
```

```
boot.ci(bootResults, type = "bca", index = 3)
```

```
## BOOTSTRAP CONFIDENCE INTERVAL CALCULATIONS
## Based on 2000 bootstrap replicates
## 
## CALL : 
## boot.ci(boot.out = bootResults, type = "bca", index = 3)
## 
## Intervals : 
## Level       BCa          
## 95%   (-0.8269,  0.7285 )  
## Calculations and Intervals on Original Scale
```

```
boot.ci(bootResults, type = "bca", index = 4)
```

```
## BOOTSTRAP CONFIDENCE INTERVAL CALCULATIONS
## Based on 2000 bootstrap replicates
## 
## CALL : 
## boot.ci(boot.out = bootResults, type = "bca", index = 4)
## 
## Intervals : 
## Level       BCa          
## 95%   (-0.9363,  0.3866 )  
## Calculations and Intervals on Original Scale
```

```
boot.ci(bootResults, type = "bca", index = 5)
```

```
## BOOTSTRAP CONFIDENCE INTERVAL CALCULATIONS
## Based on 2000 bootstrap replicates
## 
## CALL : 
## boot.ci(boot.out = bootResults, type = "bca", index = 5)
## 
## Intervals : 
## Level       BCa          
## 95%   (-0.352,  0.172 )  
## Calculations and Intervals on Original Scale
```

```
boot.ci(bootResults, type = "bca", index = 6)
```

```
## BOOTSTRAP CONFIDENCE INTERVAL CALCULATIONS
## Based on 2000 bootstrap replicates
## 
## CALL : 
## boot.ci(boot.out = bootResults, type = "bca", index = 6)
## 
## Intervals : 
## Level       BCa          
## 95%   (-3.250,  6.619 )  
## Calculations and Intervals on Original Scale
```

```
boot.ci(bootResults, type = "bca", index = 7)
```

```
## BOOTSTRAP CONFIDENCE INTERVAL CALCULATIONS
## Based on 2000 bootstrap replicates
## 
## CALL : 
## boot.ci(boot.out = bootResults, type = "bca", index = 7)
## 
## Intervals : 
## Level       BCa          
## 95%   ( 4.646, 14.105 )  
## Calculations and Intervals on Original Scale
```

```
boot.ci(bootResults, type = "bca", index = 8)
```

```
## BOOTSTRAP CONFIDENCE INTERVAL CALCULATIONS
## Based on 2000 bootstrap replicates
## 
## CALL : 
## boot.ci(boot.out = bootResults, type = "bca", index = 8)
## 
## Intervals : 
## Level       BCa          
## 95%   ( 0.1789,  1.1377 )  
## Calculations and Intervals on Original Scale
```

```
boot.ci(bootResults, type = "bca", index = 9)
```

```
## BOOTSTRAP CONFIDENCE INTERVAL CALCULATIONS
## Based on 2000 bootstrap replicates
## 
## CALL : 
## boot.ci(boot.out = bootResults, type = "bca", index = 9)
## 
## Intervals : 
## Level       BCa          
## 95%   (-1.0680,  0.7296 )  
## Calculations and Intervals on Original Scale
```

```
boot.ci(bootResults, type = "bca", index = 10)
```

```
## BOOTSTRAP CONFIDENCE INTERVAL CALCULATIONS
## Based on 2000 bootstrap replicates
## 
## CALL : 
## boot.ci(boot.out = bootResults, type = "bca", index = 10)
## 
## Intervals : 
## Level       BCa          
## 95%   (-1.6032,  0.0635 )  
## Calculations and Intervals on Original Scale
```

```
# confidence intervals in fit42
round(confint(fit42), 2)
```

```
##                  2.5 % 97.5 %
## (Intercept)      -0.41   7.21
## Age              -0.07   0.12
## Gender           -0.76   0.70
## EDU              -0.98   0.39
## TECH_KN          -0.38   0.20
## CLASS1           -3.70   6.53
## CLASS2            4.63  14.17
## DES_ST_RA         0.09   1.16
## CLASS1:DES_ST_RA -1.06   0.80
## CLASS2:DES_ST_RA -1.65   0.06
```

```
# Compare the control-only model and the regression model with rational decision style as a moderator
anova(fit12, fit42)
```

```
## Analysis of Variance Table
## 
## Model 1: CORRECT_ALL ~ Age + Gender + EDU + TECH_KN + CLASS
## Model 2: CORRECT_ALL ~ Age + Gender + EDU + TECH_KN + CLASS * DES_ST_RA
##   Res.Df    RSS Df Sum of Sq      F  Pr(>F)  
## 1    183 971.74                              
## 2    180 934.71  3    37.032 2.3771 0.07148 .
## ---
## Signif. codes:  0 '***' 0.001 '**' 0.01 '*' 0.05 '.' 0.1 ' ' 1
```

```
# Rational decision-making style as moderator plot
p3 <- ggplot(Experiment, aes(x = DES_ST_RA, y = CORRECT_ALL, group = CLASS)) +
  geom_smooth(size=1.5,aes(linetype = CLASS, color = CLASS), method = 'lm', se = F) +
  theme_classic()+
  scale_linetype_manual(values=c("dotted", "dashed", "solid"), labels = c("Control", "Tags-based", "Taxonomy-based")) +
  scale_color_manual(values = c('#000000', '#0072B2', '#C4961A'), labels = c("Control", "Tags-based", "Taxonomy-based")) +
  theme(legend.position="top",
        axis.title.x = element_text(size=14),
        axis.text.x  = element_text(size=14),
        axis.title.y = element_text(size=14),
        axis.text.y  = element_text(size=14),
        legend.title = element_text(size=14),
        legend.text = element_text(size=12)) +
  xlab("Rational decision style") +
  ylab("Selection accuracy") +
  guides(color = guide_legend("Decision aids"), linetype = guide_legend("Decision aids"))

p3
```

```
# ggsave(filename = "p3.png", width = 6.3, height = 4, dpi = 300)
```

```
# hierarchical table for fit11, fit12, fit22, fit23, f24 (testing moderating effect of decision style).
 stargazer(fit11, fit12, fit41, fit42, title = "Hierarchical Regression (H4a, H4b)", type="text",
            digits = 2,
           digits.extra = 2,
           star.char = c("+", "*", "**", "***"),
           star.cutoffs = c(.1, .05, .01, .001),
           notes= "+P<0.1; *P<0.05; **P<0.01; ***P<0.001", 
           notes.append = FALSE)
```

```
## 
## Hierarchical Regression (H4a, H4b)
## ===========================================================================================================
##                                                       Dependent variable:                                  
##                     ---------------------------------------------------------------------------------------
##                                                           CORRECT_ALL                                      
##                            (1)                  (2)                    (3)                    (4)          
## -----------------------------------------------------------------------------------------------------------
## Age                        0.01                 0.01                   0.01                   0.03         
##                           (0.07)               (0.05)                 (0.05)                 (0.05)        
##                                                                                                            
## Gender                    -0.32                -0.02                   0.06                  -0.03         
##                           (0.52)               (0.37)                 (0.38)                 (0.37)        
##                                                                                                            
## EDU                       -0.09                -0.20                  -0.21                  -0.30         
##                           (0.49)               (0.35)                 (0.35)                 (0.35)        
##                                                                                                            
## TECH_KN                    0.07                -0.09                  -0.07                  -0.09         
##                           (0.20)               (0.15)                 (0.15)                 (0.15)        
##                                                                                                            
## CLASS1                                         0.75+                   0.91                   1.42         
##                                                (0.41)                 (1.33)                 (2.59)        
##                                                                                                            
## CLASS2                                        5.11***                4.78***                9.40***        
##                                                (0.41)                 (1.33)                 (2.42)        
##                                                                                                            
## DES_ST_IN                                                             -0.21                                
##                                                                       (0.26)                               
##                                                                                                            
## CLASS1:DES_ST_IN                                                      -0.05                                
##                                                                       (0.38)                               
##                                                                                                            
## CLASS2:DES_ST_IN                                                       0.10                                
##                                                                       (0.37)                               
##                                                                                                            
## DES_ST_RA                                                                                    0.62*         
##                                                                                              (0.27)        
##                                                                                                            
## CLASS1:DES_ST_RA                                                                             -0.13         
##                                                                                              (0.47)        
##                                                                                                            
## CLASS2:DES_ST_RA                                                                             -0.79+        
##                                                                                              (0.43)        
##                                                                                                            
## Constant                 8.74***              6.94***                7.49***                 3.40+         
##                           (1.64)               (1.19)                 (1.43)                 (1.93)        
##                                                                                                            
## -----------------------------------------------------------------------------------------------------------
## Observations               190                  190                    190                    190          
## R2                        0.003                 0.50                   0.50                   0.52         
## Adjusted R2               -0.02                 0.48                   0.48                   0.49         
## Residual Std. Error  3.23 (df = 185)      2.30 (df = 183)        2.31 (df = 180)        2.28 (df = 180)    
## F Statistic         0.15 (df = 4; 185) 30.33*** (df = 6; 183) 20.23*** (df = 9; 180) 21.47*** (df = 9; 180)
## ===========================================================================================================
## Note:                                                                 +P<0.1; *P<0.05; **P<0.01; ***P<0.001
```

```
# hierarchical table for fit11, fit12, fit22, fit23, f24 (testing moderating effect of decision style) as word file
# stargazer(fit11, fit12, fit41, fit42, title = "Hierarchical Regression (H4a, H4b)", type="html",
#           digits = 2,
#           digits.extra = 2,
#           star.char = c("+", "*", "**", "***"),
#           star.cutoffs = c(.1, .05, .01, .001),
#           notes= "+P<0.1; *P<0.05; **P<0.01; ***P<0.001", 
#           out="regression04.doc",
#           notes.append = FALSE,
#           single.row = T)

# Detailed analysis of moderation effect of rational decision style
# Using 1 SD above and below mean to test the interaction
# create interaction 
sd(Experiment$DES_ST_RA)
```

```
## [1] 0.9062701
```

```
mean(Experiment$DES_ST_RA)
```

```
## [1] 5.468421
```

```
Experiment$DES_ST_RA1 <- round((Experiment$DES_ST_RA - sd(Experiment$DES_ST_RA)), 2)
Experiment$DES_ST_RA2 <- round((Experiment$DES_ST_RA + sd(Experiment$DES_ST_RA)), 2)

# Subset with control and taxonomy
Experimentsub <- subset(Experiment, TREATMENT == "Control" | TREATMENT == "Taxonomy")

Experimentsub$CLASS <- as.factor(Experimentsub$CLASS)

fit31 <- lm(CORRECT_ALL ~ DES_ST_RA1 * CLASS, data = Experimentsub)
summary(fit31)
```

```
## 
## Call:
## lm(formula = CORRECT_ALL ~ DES_ST_RA1 * CLASS, data = Experimentsub)
## 
## Residuals:
##     Min      1Q  Median      3Q     Max 
## -6.6799 -1.6103  0.3561  1.9778  4.6926 
## 
## Coefficients:
##                   Estimate Std. Error t value Pr(>|t|)    
## (Intercept)         3.9904     1.2930   3.086   0.0025 ** 
## DES_ST_RA1          0.5956     0.2828   2.106   0.0372 *  
## CLASS2              8.5707     2.0994   4.082 7.93e-05 ***
## DES_ST_RA1:CLASS2  -0.7758     0.4484  -1.730   0.0861 .  
## ---
## Signif. codes:  0 '***' 0.001 '**' 0.01 '*' 0.05 '.' 0.1 ' ' 1
## 
## Residual standard error: 2.388 on 124 degrees of freedom
## Multiple R-squared:  0.5466, Adjusted R-squared:  0.5356 
## F-statistic: 49.82 on 3 and 124 DF,  p-value: < 2.2e-16
```

```
betas.lm(fit31)
```

```
##                         beta    se.beta
## DES_ST_RA1         0.1652456 0.07844632
## CLASS2             1.2279654 0.30079033
## DES_ST_RA1:CLASS2 -0.5368014 0.31025498
```

```
fit32 <- lm(CORRECT_ALL ~ DES_ST_RA * CLASS, data = Experimentsub)
summary(fit32)
```

```
## 
## Call:
## lm(formula = CORRECT_ALL ~ DES_ST_RA * CLASS, data = Experimentsub)
## 
## Residuals:
##     Min      1Q  Median      3Q     Max 
## -6.6799 -1.6103  0.3561  1.9778  4.6926 
## 
## Coefficients:
##                  Estimate Std. Error t value Pr(>|t|)    
## (Intercept)        3.4484     1.5445   2.233 0.027369 *  
## DES_ST_RA          0.5956     0.2828   2.106 0.037178 *  
## CLASS2             9.2767     2.5004   3.710 0.000311 ***
## DES_ST_RA:CLASS2  -0.7758     0.4484  -1.730 0.086084 .  
## ---
## Signif. codes:  0 '***' 0.001 '**' 0.01 '*' 0.05 '.' 0.1 ' ' 1
## 
## Residual standard error: 2.388 on 124 degrees of freedom
## Multiple R-squared:  0.5466, Adjusted R-squared:  0.5356 
## F-statistic: 49.82 on 3 and 124 DF,  p-value: < 2.2e-16
```

```
betas.lm(fit32)
```

```
##                        beta    se.beta
## DES_ST_RA         0.1652456 0.07844632
## CLASS2            1.3291200 0.35823997
## DES_ST_RA:CLASS2 -0.6352225 0.36713938
```

```
fit33 <- lm(CORRECT_ALL ~ DES_ST_RA2 * CLASS, data = Experimentsub)
summary(fit33)
```

```
## 
## Call:
## lm(formula = CORRECT_ALL ~ DES_ST_RA2 * CLASS, data = Experimentsub)
## 
## Residuals:
##     Min      1Q  Median      3Q     Max 
## -6.6799 -1.6103  0.3561  1.9778  4.6926 
## 
## Coefficients:
##                   Estimate Std. Error t value Pr(>|t|)    
## (Intercept)         2.9063     1.7977   1.617 0.108484    
## DES_ST_RA2          0.5956     0.2828   2.106 0.037178 *  
## CLASS2              9.9827     2.9033   3.438 0.000797 ***
## DES_ST_RA2:CLASS2  -0.7758     0.4484  -1.730 0.086084 .  
## ---
## Signif. codes:  0 '***' 0.001 '**' 0.01 '*' 0.05 '.' 0.1 ' ' 1
## 
## Residual standard error: 2.388 on 124 degrees of freedom
## Multiple R-squared:  0.5466, Adjusted R-squared:  0.5356 
## F-statistic: 49.82 on 3 and 124 DF,  p-value: < 2.2e-16
```

```
betas.lm(fit33)
```

```
##                         beta    se.beta
## DES_ST_RA2         0.1652456 0.07844632
## CLASS2             1.4302746 0.41597244
## DES_ST_RA2:CLASS2 -0.7343868 0.42445334
```

```
# Create new variable for rational decision-making style based on the actual mean/standard deviation in our data set

DES_ST_RA.SD <- c(mean(Experimentsub$DES_ST_RA)-sd(Experimentsub$DES_ST_RA),
                  mean(Experimentsub$DES_ST_RA),
                  mean(Experimentsub$DES_ST_RA)+sd(Experimentsub$DES_ST_RA))

DES_ST_RA.SD <- round(DES_ST_RA.SD, 2)
DES_ST_RA.SD
```

```
## [1] 4.50 5.47 6.44
```

```
fit32 <- lm(CORRECT_ALL ~ CLASS * DES_ST_RA, data = Experimentsub)
library(effects)
```

```
## Use the command
##     lattice::trellis.par.set(effectsTheme())
##   to customize lattice options for effects plots.
## See ?effectsTheme for details.
```

```
Inter.SD <- effect(c("CLASS * DES_ST_RA"), fit32,
                   xlevels=list(DES_ST_RA=c(4.5, 5.47, 6.44),
                                CLASS=c(0, 2))) 

Inter.SD <- as.data.frame(Inter.SD)
head(Inter.SD)
```

```
##   CLASS DES_ST_RA       fit        se     lower     upper
## 1     0      4.50  6.128749 0.3848611  5.367001  6.890497
## 2     2      4.50 11.914160 0.4811331 10.961863 12.866457
## 3     0      5.47  6.706517 0.3000787  6.112578  7.300457
## 4     2      5.47 11.739361 0.3010866 11.143426 12.335296
## 5     0      6.44  7.284286 0.4271266  6.438883  8.129689
## 6     2      6.44 11.564562 0.4215915 10.730114 12.399009
```

```
# Create a factor of the variable used in the interaction                   
Inter.SD$DES_ST_RA.D <- factor(Inter.SD$DES_ST_RA,
                               levels=c(4.5, 5.47, 6.44),
                               labels=c("-1SD", "Mean", "+1SD"))

# Create a factor of the variable used in the interaction 
Inter.SD$CLASS.D <- factor(Inter.SD$CLASS,
                           levels=c(0, 2),
                           labels=c("No decision aid", "Taxonomy-based"))

head(Inter.SD)
```

```
##   CLASS DES_ST_RA       fit        se     lower     upper DES_ST_RA.D
## 1     0      4.50  6.128749 0.3848611  5.367001  6.890497        -1SD
## 2     2      4.50 11.914160 0.4811331 10.961863 12.866457        -1SD
## 3     0      5.47  6.706517 0.3000787  6.112578  7.300457        Mean
## 4     2      5.47 11.739361 0.3010866 11.143426 12.335296        Mean
## 5     0      6.44  7.284286 0.4271266  6.438883  8.129689        +1SD
## 6     2      6.44 11.564562 0.4215915 10.730114 12.399009        +1SD
##           CLASS.D
## 1 No decision aid
## 2  Taxonomy-based
## 3 No decision aid
## 4  Taxonomy-based
## 5 No decision aid
## 6  Taxonomy-based
```

```
# Plot of SD analysis
p4 <- ggplot(data=Inter.SD, aes(x = CLASS.D, y = fit, group = DES_ST_RA.D))+
  geom_line(size=1.5, aes(linetype = DES_ST_RA.D, color = DES_ST_RA.D))+
  scale_linetype_manual(values=c("solid", "dotted", "dashed" )) +
  scale_x_discrete(expand = c(0, 0.2)) +
  theme_classic() +
  scale_color_manual(values = c('#0072B2','#000000', '#C4961A')) +
  theme(legend.position="top",
        axis.title.x = element_text(size=14),
        axis.text.x  = element_text(size=14),
        axis.title.y = element_text(size=14),
        axis.text.y  = element_text(size=14),
        legend.title = element_text(size=14),
        legend.text = element_text(size=12)) +
  ylab("Selection accuracy")+
  xlab("Decision aids") +
  guides(color = guide_legend("Rational decision style"), linetype = guide_legend("Rational decision style"))
 
p4
```

```
# ggsave(filename = "p4.png", width = 6.3, height = 4, dpi = 300)
# ggarrange(p3,p4)
# ggsave(filename = "p34.png", width = 11, height = 4.5, dpi = 300)
```
