# Supplementary material for: Experimental materials comparing individual performance implications of two decision aids: Taxonomy and tags
Source: MethodsX. 2020 Nov 17;7:101133. doi: 10.1016/j.mex.2020.101133 (PMC7701260; doi:10.1016/j.mex.2020.101133)
Supplement: Supplementary file 3 [file mmc3.docx]

**The Creation of the Tags used in the Experiment**

1. Open Card Sorting Procedure

The repeated single-criterion sorts suggested by Rugg and Mcgeorge (1997) were followed in the sorting process. In repeated single-criterion sorts, participants were asked to sort the cards based on a single criterion (i.e., viewpoint) each time [1]. As novices intend to sort entities from lower level categories [2], it is more flexible and easier for novices to handle when sorting techniques based on only one viewpoint [1]. In the sorting process, participants were, at first, given instruction of card sorting. Subsequently, they started to sort design techniques into different categories and gave labels to the categories. Finally, they were asked to fill out a questionnaire as input for collecting their demographic information and the perceived confidence of the sorting result.

**Participants**

With a purpose of understanding how design novices classify design techniques, an open card sorting was conducted with 40 students studying computer science, information systems, industrial engineering and management with an average age of 22 years (SD = 2.2) (23 male, 17 female). The students had some basic knowledge of design, and they could be potential consumers of design techniques in their future job but did not have any real-world practical experiences when conducting the experiment. Thus, it was reasonable to consider students as novices and conduct open card sorting with them. In the open card sorting exercise, in order to avoid bias from a single person (cf., Burnay 2016), two students were randomly assigned to a team, and 20 teams were built. Two participants were asked to sort the cards together. Each team sorted cards without interruptions or influences from other teams.

**Materials**

As the card sorting process is suggested to include 30-100 cards [4], 70 self-explained small filing cards (i.e., 70 design techniques^[[1]](#footnote-1)^) were used for sorting. Several envelopes were provided for collecting created categories. The suggestion of Rugg and McGeorge (1997) were followed to prepare small self-explained filing cards (Figure 1).


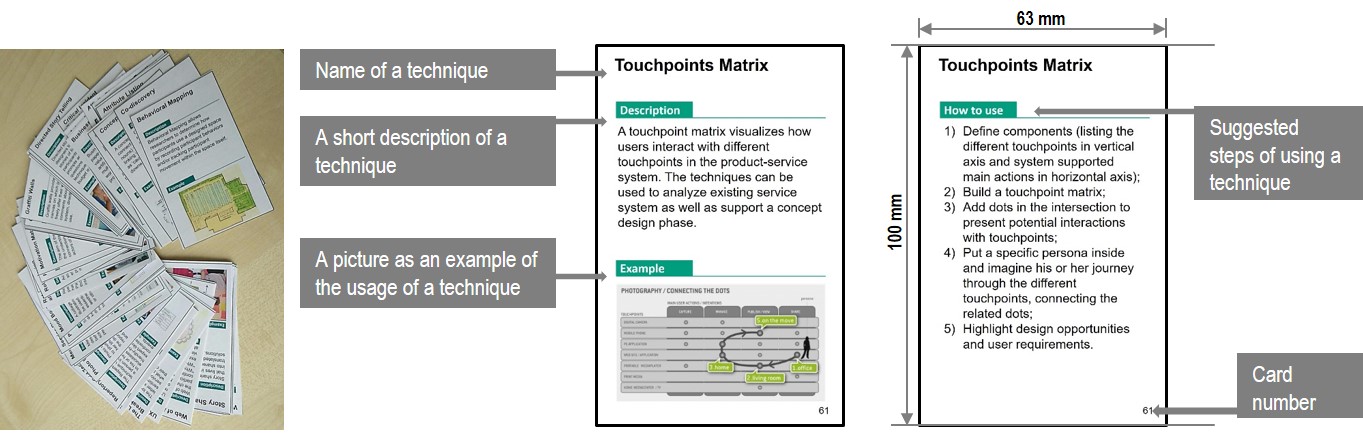


Note: The content and picture on the card were from Brugnoli (2011).

*Figure 1. An example of a self-explained card*

The size of each card was 63mm x 100mm to make sure the cards were easy to handle. Each card included five main information pieces. The front side included the name of the design technique, a short description of the design technique, and an example or a scenario of the design technique. The back side described a brief instruction on how to use the design technique. Both sides had a number of the card for the convenience of data collection. In order to collect the sorted cards and the created categories, participants were asked to put the cards that belong to the same category into an envelope and label each category and/or give an explanation to each category. The viewpoint that they based on for categorizing and the team number also needed to be written on the envelopes.

**Sorting Steps**

The open card sorting process was divided into three steps: reading a short instruction of card sorting, sorting cards into categories, and answering a questionnaire [1]. *First,* what open card sorting is and how to sort cards was explained. In order not to bias participants, sorting examples from zoology was provided in the instruction. For instance, polar bears and koala bears can be classified as Mammalia based on the viewpoints of morphology. However, polar bear and koala bear belong to two categories, carnivore and herbivore, based on the viewpoint of eating patterns. Such examples were used to explain that the participants can create the design techniques based on different viewpoints and there is no wrong classification. *Second,* each team read the information on each card and sorted cards into categories based on their understandings of design techniques. The participants were expected to sort design techniques based on only one viewpoint and create no less than three and no more than nine categories [1,6]. Each team put sorted cards into envelopes and wrote a label and/or a short description for each category on each envelope. If some cards could not be sorted into a category, students could put them into an envelope called “not applicable.” *Third,* after each team finished the sorting, each participant was asked to fill out a questionnaire independently. The questionnaire was used for collecting demographic information, experiences of design projects, and the confidence level of the card sorting result from the sorting session. All participants were very confident about their sorting results. The open card sorting session for each team lasted between 45 to 60 minutes.

1. Result

After collecting the outcomes from open card sorting, the exploratory analysis approach was followed to analyze the result [4]. *First,* the viewpoints that novices used as the basis for categorizing design techniques were analyzed and aggregated. *Second,* categories from the originally created categories with similar meanings were standardized. In the open card sorting process, there were no pre-defined categories. All the categories were created by the participants themselves. As 20 teams participated in the sorting exercise, 20 groups of categories were created. Different teams might create the same categories or use different labels for the same categories. Before comparing categories created by novices with the categories in the expert-based classifications, the same categories, and categories with similar meanings were merged to create standardized categories [4,7]. *Third,* techniques were assigned into standardized categories. In the collected result, besides the created categories, the classified techniques under each category were also presented. An overview of the assigned design techniques into different categories was presented. In order to have a deep understanding of the overlap between design techniques and standardized categories, the percentage of the teams that agreed to the assignment of each design technique in the standardized categories were computed, which depicted the overall agreement of the allocation of design techniques from novices.

Viewpoints

The open card sorting result showed that 20 teams created 110 categories (excluding the category “not applicable”) based on 11 different viewpoints. The used viewpoints were summarized in Table 1. More than 63.6% of the participants sorted cards by combining two or more viewpoints, even though students were told to sort the cards based on only one viewpoint. Take team 5 as an example; team 5 created five categories which include “use diagrams to visualize results,” “only designer participation,” “pre-experience scenarios to solve problems,” “collaboration between user and designer,” and “user-oriented”. These five categories described two viewpoints: participants and activity types. For most of the participants, the combined viewpoint might refer to a unique viewpoint. The most mentioned viewpoint was purpose which was considered as a single viewpoint by three teams and appeared in combined viewpoints in eight teams. The most used combined viewpoint was purpose and activity type.

*Table 1. Viewpoints that novices used to categorize design techniques*

| Team No. | Viewpoint | Meaning |
| --- | --- | --- |
| 3 | Activity type | The types that the activities (e.g., observation, simulation) are conducted when using the technique. |
| 15 | Phase | The phase that the techniques can be used in the design process. |
| 7, 11 | Duration | The time length required when using the techniques |
| 4, 9, 10, 12 | Purpose | The reason for using the techniques |
| 1 | Phase and activity type | Combine the phases with activity types (e.g., collaborative work, draw chart) when using the techniques |
| 8 | Duration and Participant | The time length that different participants need when using the techniques |
| 6 | Participant and activity type | The activity types of the involved participants |
| 5 | Participant and purpose | The participants’ purposes when using a design technique |
| 16, 17, 19 | Participant, purpose, and activity type | Combination of participant, purpose and activity type |
| 2, 13, 14, 18 | Purpose and activity type | Combination of purpose and activity type |
| 20 | Representation of the technique | The superficial appearance of a technique (e.g., bubble chart) |

Categories of Design Techniques

When looking at the created categories, the minimum number of the categories was created by team 11 with three categories based on the viewpoint of duration. The maximum number of the categories was created by team 19 with nine categories based on the viewpoints of participant, purpose, and activity type. The average number of created categories was 5.5. As some of the created categories had the same name and some of them had similar meaning, standardized categories were at first created to aggregate the sorting result from novices, which could also help with analyzing created categories and comparing with the experts’ created categories. Three researchers went through all categories and created 16 standardized categories from 110 original categories (Table 2).

The 16 standardized categories were used by at least two teams. Some categories were built by the single team, for instance, user simulation, user participation (user group), flexible time length, feedback collection (offline), feedback collection (online), user simulation, etc. To analyze the categories created by only one team could also be interesting, but such categories could not present the commonalities between novice. Thus, the standardized categories were mentioned by at least two teams. Jaccard’s Coefficient was used to calculate the agreement between teams in the standardized categories because Jaccard’s Coefficient is usually used in the analysis of categories from open card sorting [8,9]. Compared with the example of standardized open card sorting categories provided in Spencer (2009), the Jaccard’s Coefficient showed that the standardized categories could be used for the further analysis of the assignment of design techniques. Around half of the participants created the following categories: user research, idea generation, and information organization. These three categories belonged to the viewpoint purpose of using design techniques.

*Table 2. Standardized categories and examples of assigned design techniques*

| Standardized category | #Teams | Jaccard’s Coefficient | Standardized category | #Teams | Jaccard’s Coefficient |
| --- | --- | --- | --- | --- | --- |
| User research | 11 | 0.370 | Product evaluation | 3 | 0.400 |
| Idea generation | 10 | 0.340 | Collaboration with stakeholders | 3 | 0.405 |
| Information organization | 9 | 0.372 | User participation | 3 | 0.489 |
| Feedback collection | 5 | 0.411 | Short-term duration | 3 | 0.661 |
| Prototype evaluation | 5 | 0.350 | Long-term duration | 3 | 0.474 |
| Prototyping | 5 | 0.400 | UX evaluation | 2 | 0.647 |
| Evaluation | 4 | 0.414 | Mid-term duration | 2 | 0.528 |
| Expert participation | 4 | 0.551 | Relationship and dependency | 2 | 0.536 |
| *Note: #Teams means the numbers of teams considered the standardized category.* | | | | | |

Assignment of Design Techniques to Categories

The analysis of the standardized categories shows that approximately half of the teams created user research, idea generation, and information organization. Card sorting participants assigned many techniques in the three categories (62, 55, 43 techniques separately). But not all the assigned techniques had a high percentage of the overlap between design techniques and standardized categories. Thus, the percentage of participants that assigned a design technique to a standardized category were computed. In total, there were 70 design techniques and 16 standardized categories. Take actors mapping as an example, 30% of the 20 teams (i.e., six teams) put the design technique actors mapping in the standardized category information organization. As there were nine teams who created the standardized category information organization (Table 2), which meant six out of nine teams allocated actors mapping to standardized category information organization. Thus, actors mapping could be assigned into the category information organization. The entire information was analyzed by following this procedure. When there were more than half of the participants who used the standard categories assigned a technique into the specific standardized categories, the categories were highlighted in Table 3. As the participants created categories from different viewpoints, the standardized categories were not mutually exclusive. For example, the design technique affinity diagramming was classified into two standardized categories idea generation (eight out of ten teams) and information organization (five out of nine teams).

*Table 3. Overlaps between standardized categories and design techniques.*

| Card name | User Research | Idea generation | Information Organization | Feedback Collection | Prototype evaluation | Prototyping | Evaluation | Expert participation | Product Evaluation | Collaboration with stakeholders | User participation | Short term duration | Long term duration | UX Evaluation | Mid-term duration | Relationships and Dependencies |
| --- | --- | --- | --- | --- | --- | --- | --- | --- | --- | --- | --- | --- | --- | --- | --- | --- |
| 3-12-3 Brainstorming | 5% | 45% | 10% |  |  |  | 5% | 15% |  | 5% |  | 10% |  |  |  |  |
| 3E- Expressing Emotion and Experience | 26% |  |  | 21% | 16% |  | 5% |  |  |  |  | 16% |  | 5% |  | 5% |
| 6-3-5 Brainwriting | 5% | 45% | 10% |  |  |  | 5% | 15% |  | 5% |  | 10% |  |  |  |  |
| A/B Testing | 16% |  |  | 16% | 16% | 5% | 16% |  |  |  |  |  | 5% |  | 5% |  |
| Actors Mapping | 20% | 10% | 30% |  |  |  |  |  |  | 5% | 5% | 5% | 5% |  | 5% | 10% |
| Affinity Diagramming |  | 40% | 25% |  |  |  |  | 15% |  |  |  |  | 5% |  | 5% | 5% |
| Attribute Listing | 10% | 10% | 20% | 5% |  |  | 5% | 15% | 5% |  |  | 10% |  |  |  |  |
| Behavioral Mapping | 40% |  | 20% | 5% |  |  | 5% |  |  |  |  |  | 5% | 5% | 5% | 5% |
| Bodystorming |  | 16% |  | 5% | 16% | 5% | 5% | 16% |  |  |  | 11% |  | 11% |  | 5% |
| Business Origami | 5% | 40% |  |  |  |  |  | 20% |  | 10% |  | 10% |  |  |  | 5% |
| Closed Card Sorting | 15% | 25% | 15% | 5% |  |  | 5% | 5% |  |  |  | 15% |  |  |  | 5% |
| Co-Discovery | 30% |  |  | 10% | 10% |  | 10% |  |  |  | 5% | 5% | 5% | 5% | 5% | 5% |
| Cognitive Mapping |  | 25% | 35% | 5% | 5% |  |  | 10% |  |  |  | 5% | 5% |  |  |  |
| Cognitive Walkthrough | 5% | 5% | 11% |  | 11% | 16% | 11% | 21% |  |  |  | 5% |  | 5% |  |  |
| Collaborative Sketching | 11% | 11% |  |  | 5% | 21% |  |  |  | 5% | 16% | 16% |  |  |  | 5% |
| Concept Mapping |  | 30% | 35% | 5% | 5% |  |  | 10% |  |  | 5% | 10% |  |  |  |  |
| Content Inventory & Auditing | 5% |  | 20% | 10% |  | 5% | 10% | 5% | 5% |  |  | 5% | 5% |  |  |  |
| Contextual Laddering | 35% | 5% |  | 15% | 5% |  |  |  |  |  | 10% | 15% |  |  |  | 5% |
| Critical Incident Technique | 32% | 11% | 5% |  |  |  | 5% | 5% |  | 5% | 5% |  | 11% |  |  | 5% |
| Concurrent Think-Aloud | 16% |  |  | 21% | 5% | 5% | 16% |  |  |  | 11% | 16% |  |  |  | 5% |
| Desirability Testing with Product Research Cards | 15% | 5% | 5% | 20% |  | 5% | 10% | 5% |  |  | 5% | 5% | 10% | 5% |  | 5% |
| Diary Studies | 42% | 11% |  | 5% |  |  |  | 11% |  |  |  |  | 16% |  |  | 5% |
| Directed Storytelling | 47% | 11% |  |  |  |  | 5% |  |  |  | 11% | 11% |  |  | 5% | 5% |
| Experience Clip | 30% |  | 5% | 10% | 5% |  | 5% | 5% |  |  |  | 5% | 5% | 5% |  | 5% |
| Experience Prototyping | 5% | 5% |  |  | 16% | 26% |  |  |  |  | 5% | 11% |  | 11% | 5% |  |
| Eye-tracking | 40% |  |  | 5% |  |  | 10% |  | 10% |  | 5% | 10% |  |  |  |  |
| Flexible Modeling | 21% | 5% | 5% | 5% | 5% | 21% |  |  |  |  | 11% | 16% |  |  |  |  |
| Fly-on-the-Wall Observation | 55% | 10% |  |  |  |  |  | 5% |  |  |  |  | 5% |  | 5% | 5% |
| Graffiti Walls | 25% | 20% |  | 10% |  |  | 5% |  |  | 5% | 5% | 10% | 5% |  |  | 5% |
| Heuristic Evaluation | 11% |  | 5% | 16% | 21% | 5% | 5% | 21% |  |  |  | 5% | 5% |  |  |  |
| Kano Analysis | 35% | 5% | 5% |  | 10% |  | 10% |  |  |  |  | 10% | 5% |  |  | 5% |
| LEGO Serious Play | 5% | 26% | 5% |  |  | 11% |  | 11% |  | 5% |  | 16% |  | 11% |  |  |
| Mental Model Diagramming | 10% | 25% | 20% |  |  |  | 15% |  | 5% |  | 5% |  | 5% |  |  |  |
| Mind Mapping | 5% | 40% | 25% |  |  |  |  | 10% |  |  |  | 10% |  |  |  | 5% |
| Mood Boards | 30% | 20% | 10% |  |  |  |  | 5% |  |  | 10% | 15% |  |  |  |  |
| Motivation Matrix | 5% | 25% | 25% | 5% |  |  |  | 5% |  | 5% |  | 15% |  |  |  | 5% |
| Offering Mapping | 10% | 20% | 30% |  |  | 5% |  | 10% |  |  |  | 10% |  | 5% |  | 5% |
| Open Card Sorting | 25% | 20% | 10% | 5% |  |  | 5% |  |  |  | 5% | 10% |  |  | 5% | 5% |
| Parallel Prototyping | 26% | 5% | 5% |  | 11% | 21% |  | 5% |  |  |  |  | 11% |  | 5% |  |
| Personas | 20% | 10% | 15% |  | 10% |  |  | 15% |  |  |  |  | 5% | 10% | 5% | 5% |
| Photo Diary | 40% | 10% |  | 5% |  |  |  | 5% |  |  | 10% | 5% | 10% |  |  | 5% |
| Photo Elicitation Interviewing | 45% | 10% |  | 5% |  |  |  |  |  |  | 10% | 10% |  |  | 5% | 5% |
| Private Camera Conversation | 21% |  |  | 16% | 21% |  | 11% |  |  |  |  | 11% |  |  |  | 5% |
| Product Experience Tracker | 15% |  | 5% | 25% | 5% |  | 10% |  | 10% |  | 5% | 5% | 10% |  |  |  |
| Repertory Grid | 16% |  | 21% | 16% | 11% |  | 11% |  |  |  |  | 11% |  |  | 5% |  |
| Retrospective Think-Aloud | 11% |  |  | 26% | 11% |  | 11% |  |  |  | 16% | 11% |  |  |  | 5% |
| Roadmapping |  | 30% | 30% |  | 5% |  |  | 15% |  |  |  |  | 5% |  | 5% |  |
| Role-Playing | 30% | 10% |  |  | 5% |  |  | 15% |  |  |  | 5% |  | 10% |  | 5% |
| Scenarios | 30% | 15% | 10% |  | 5% |  |  | 10% |  | 5% |  | 10% |  | 5% |  | 5% |
| Sentence Completion | 25% | 5% |  | 15% | 5% |  | 5% |  |  |  | 10% | 10% |  | 5% | 5% | 5% |
| Service Blueprints | 30% | 5% | 20% |  | 5% | 5% |  |  |  |  |  | 10% | 5% |  |  |  |
| Shadowing | 50% | 10% |  | 5% |  |  |  |  |  |  | 5% |  | 10% |  |  | 5% |
| Speed Dating | 15% |  |  | 5% | 15% | 5% | 10% |  | 5% |  | 10% | 10% |  |  | 5% |  |
| Stakeholder Maps | 5% | 25% | 25% | 5% |  |  |  | 5% |  | 5% | 5% | 10% |  |  |  | 10% |
| Stakeholder Walkthrough | 5% | 5% |  | 5% | 11% | 5% | 11% |  |  | 16% | 11% | 16% |  | 5% |  | 5% |
| Storyboards | 20% | 35% | 10% |  | 5% |  |  | 10% |  |  |  | 10% |  |  |  | 5% |
| Story Sharing | 15% | 25% |  |  |  | 5% |  | 20% |  | 5% |  | 10% |  |  |  | 5% |
| Territory Maps |  | 30% | 25% |  |  |  |  | 15% |  |  |  | 10% |  |  |  |  |
| The Love Letter & Breakup Letter | 25% | 5% |  | 15% | 5% |  | 5% |  |  |  | 5% | 15% |  | 5% |  | 5% |
| Tomorrow Headlines | 25% | 30% | 5% |  | 10% |  |  |  |  |  |  | 15% |  |  |  |  |
| Touchpoints Matrix | 25% | 5% | 20% | 5% | 5% |  |  | 10% |  |  |  | 10% |  | 5% |  | 10% |
| User Journey Maps | 25% | 5% | 25% |  | 5% |  |  |  |  |  | 5% |  | 5% |  |  | 5% |
| UX Curve | 10% | 5% | 15% | 20% |  |  | 10% |  | 5% |  | 5% |  | 15% |  |  |  |
| Value Mapping |  | 20% | 30% |  |  | 5% |  | 15% |  |  |  | 10% |  |  |  | 5% |
| Value Opportunity Analysis | 5% | 21% |  | 5% |  |  | 11% | 16% | 5% |  |  | 11% |  |  |  |  |
| Web Analytics | 15% | 5% | 15% | 10% |  |  | 15% | 5% | 5% |  |  |  | 10% |  |  |  |
| Web of Abstraction | 5% | 30% | 20% |  | 5% | 5% |  | 10% |  |  |  | 10% |  |  |  |  |
| Weighted Matrix |  | 15% | 25% |  |  |  | 10% | 15% | 5% | 5% |  | 10% |  |  |  | 5% |
| Wireframing | 5% | 5% | 16% |  | 5% | 26% |  | 11% |  |  | 5% | 5% |  |  | 5% |  |
| Wizard of Oz | 26% |  |  | 11% | 16% | 11% | 5% |  |  |  | 5% | 5% |  |  | 11% | 5% |
| *Note: The percentage means the agreement of all participants on assigning a technique under a standardized category (Spencer 2009). 40 design techniques from this table were used in the experiment.* | | | | | | | | | | | | | | | | |

Reference

[1] G. Rugg, P. McGeorge, The sorting techniques: a tutorial paper on card sorts, picture sorts and item sorts, Expert Syst. 14 (1997) 80–93. https://doi.org/10.1111/1468-0394.00045.

[2] M.T.H. Chi, R. Glaser, E. Rees, Expertise in problem solving, Erlbaum, Hillsdale, NJ, 1981.

[3] C. Burnay, Are Stakeholders the Only Source of Information for Requirements Engineers ? Toward a Taxonomy of Elicitation Information Sources, ACM Trans. Manag. Inf. Syst. 7 (2016) 8:1-8:29. https://doi.org/10.1145/2965085.

[4] D. Spencer, Card Sorting: Designing Usable Categories, Louis Rosenfeld, Brooklyn, New York, 2009.

[5] G. Brugnoli, Connecting the Dots of User Experience, J. Inf. Archit. 1 (2011) 6–15. .

[6] R.C. Nickerson, U. Varshney, J. Muntermann, A method for taxonomy development and its application in information systems, Eur. J. Inf. Syst. 22 (2013) 336–359. https://doi.org/10.1057/ejis.2012.26.

[7] C. Righi, J. James, M. Beasley, D.L. Day, J.E. Fox, J. Gieber, C. Howe, L. Ruby, Card Sort Analysis Best Practices, J. Usability Stud. 8 (2013) 69–89. .

[8] K.M. Lewis, P. Hepburn, Open card sorting and factor analysis: a usability case study, Electron. Libr. 28 (2010) 401–416. https://doi.org/10.1108/02640471011051981.

[9] M. Schmettow, J. Sommer, Linking card sorting to browsing performance – are congruent municipal websites more efficient to use?, Behav. Inf. Technol. 35 (2016) 452–470. https://doi.org/10.1080/0144929X.2016.1157207.

[10] R.D. Hackathorn, J. Karimi, A Framework for Comparing Information Engineering Methods, MIS Q. 12 (1988) 203–220. https://doi.org/10.2307/248845.

[11] S. Brinkkemper, Method engineering: engineering of information systems development methods and tools, Inf. Softw. Technol. 38 (1996) 275–280. https://doi.org/10.1016/0950-5849(95)01059-9.

[12] W.J. Kettinger, J.T.C. Teng, S. Guha, Business Process Change: A Study of Methodologies, Techniques, and Tools, MIS Q. 21 (1997) 55–80. https://doi.org/10.2307/249742.

1. The 70 design techniques are selected based on the definition of methods, techniques, and tools [10–12] from the following sources: allaboutux.org (includes the methods in Vermeeren et al. (2010)); servicedesignkit.org (an application of Liu et al. (2016.)); introduced techniques in Martin and Hanington (2012). These three studies were used to compare novice- and expert-based classifications. [↑](#footnote-ref-1)
