# Supplementary material for: Experimental materials comparing individual performance implications of two decision aids: Taxonomy and tags
Source: MethodsX. 2020 Nov 17;7:101133. doi: 10.1016/j.mex.2020.101133 (PMC7701260; doi:10.1016/j.mex.2020.101133)
Supplement: Supplementary file 4 [file mmc4.docx]

**The Creation of the Taxonomy used in the Experiment**

1. Development Method

A taxonomy is a set of dimensions, and each dimension consists of a set of two or more mutually exclusive and collectively exhaustive characteristics so that each object (i.e., design technique) can be classified into one and only one characteristic for each dimension [1]. Characteristics are defined as features that reflect the similarities and differences between the classified objects [1]. Taxonomies have been widely applied in many fields, e.g., in biology and medicine. In addition, taxonomies typically reflect the structure and hierarchy of a website, which helps users to find the information they need [2]. Hence, a taxonomy that provides a clear overview of the broad spectrum of available design techniques as a starting point is needed to support the selection of design techniques.

Nickerson et al. (2013)’s method was followed to develop a taxonomy of design techniques, which provides a detailed description of the principles and steps for developing taxonomies. The specific method was chosen for four reasons. First, this method suggests developing a taxonomy by identifying a subset of objects (i.e., design techniques). Given the fact that new design techniques appear all the time, it is challenging to obtain a list of all design techniques available. Therefore, the taxonomy can be developed in an iterative manner, which extends the taxonomy as new data sources. Second, the method provides subjective and objective ending conditions. These ending conditions not only guide the development process by preventing endless iterations during the development, but also suggest when further evolutions of the taxonomy are needed. When new design techniques are introduced, which cannot be assigned to any of the existing dimensions or characteristics, new dimensions or characteristics can be added to extend the taxonomy. Third, by following this method, mutually exclusive and collectively exhaustive characteristics will be created, which give a clear overview of design techniques. Fourth, the method has been successfully applied; for example, developing a taxonomy of evaluation methods for information systems artifacts [3], and developing a taxonomy to analyze the IT value literature. To summarize, Nickerson et al. (2013)’s taxonomy development method was considered as suitable to develop the taxonomy of digital service design techniques.

1. Taxonomy Development Process

Figure 1 depicts the steps that were followed to develop the taxonomy of design techniques.


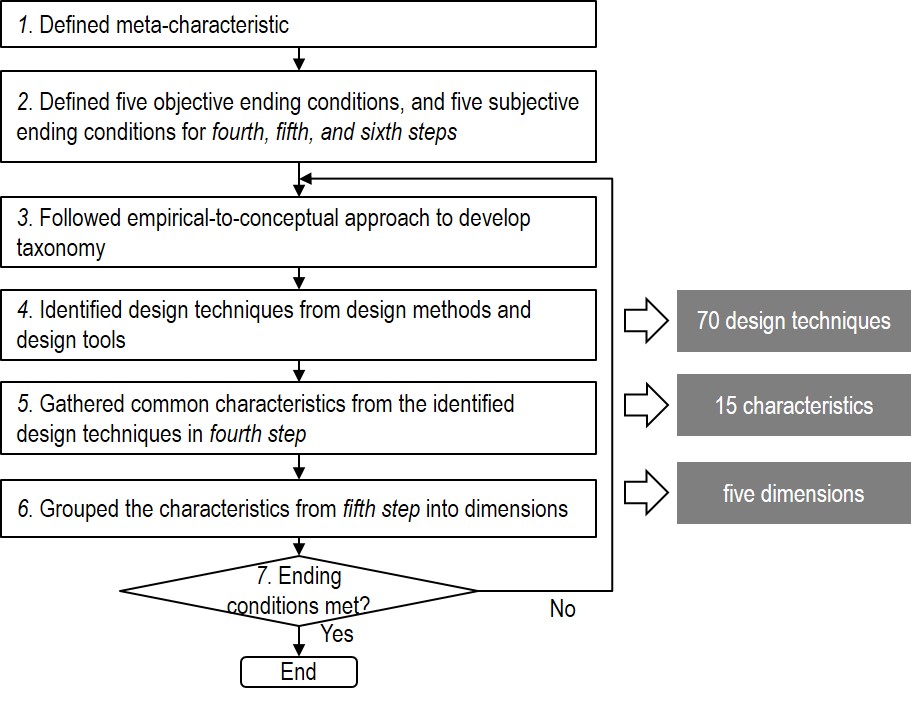


*Figure 1. Taxonomy development steps*

In the *first step*, the meta-characteristic and purpose of the taxonomy were determined, i.e., to develop a taxonomy with mutually exclusive and collectively exhaustive dimensions and characteristics that provide a comprehensive view of design techniques and to serve as a foundation for future research, supporting the selection of design techniques.

In the *second step*, the ending conditions that were used to terminate the iteration in the taxonomy development procedure were defined. Two types of ending conditions - objective ending conditions and subjective ending conditions - were formulated [1]. Objective ending conditions tested whether each dimension had mutually exclusive and collectively exhaustive characteristics. Subjective ending conditions checked whether it was a useful taxonomy from a theoretical perspective. In order to test the taxonomy with these ending conditions systematically and purposefully, the applicable ending conditions were organized in Table 1, presenting the ending conditions for each step (cf. Prat et al. 2015). The detailed steps included identifying design techniques (fourth step), identifying characteristics (fifth step), and grouping characteristics into dimensions (sixth step). To ensure that the judgment of the subjective ending conditions was unbiased, two people that were involved in the development of the taxonomy evaluated the subjective ending conditions together.

In the *third step*, the empirical-to-conceptual approach was followed to develop the taxonomy based on a systematic and detailed analysis of secondary data [4]. There are sources with plenty of design techniques and explanations for the usage of each design technique.

In the *fourth step*, design techniques were distinguished from design tools and methods because of the objective to develop a taxonomy of digital service design techniques. In the design process of digital services, the service ecologies, UX, digital transactions, and variety of activities (e.g., generating ideas, creating and evaluating prototypes) need to be considered [5–7]. The used sources should contain techniques that fit the context of digital service design. Thus, two people jointly decided the ending conditions that were used in the sources^[[1]](#footnote-1)^ from the academic and grey literature in order to choose related design techniques, which focus specifically on UI design, user-centered design, usability, and UX design. The filtering of all repeated data points results in a list of 292 design methods, design techniques, and design tools. As design methods, design techniques, and design tools were often mixed up, design techniques were distinguished from design methods (e.g., crowdsourcing, experience sampling method, evidence-based design) and design tools (e.g., AttrakDiff, AEIOU, Attrak-Work questionnaire) based on the descriptions of methods, techniques, and tools. The final list consisted of 82 design techniques. Thereafter, design techniques with similar definitions were merged. First, the technique with a more precise name that well-explained the technique was chosen (e.g., concurrent think-aloud and retrospective think-aloud were chosen instead of think-aloud). Second, if neither name was considered more precise, the design techniques that had been used most often was selected for the final list (e.g., affinity diagramming was chosen instead of KJ technique). In sum, the number of design techniques decreased to a list of 70 design techniques.

*Table 1. Ending conditions for developing the taxonomy of design techniques*

| Ending conditions | Steps |
| --- | --- |
| *Objective ending conditions* |  |
| All design techniques have been examined and cannot be merged or split. | 4 |
| At least one design technique is classified under each characteristic. | 4, 5 |
| Each characteristic is unique and cannot be repeated (no characteristic duplication). | 5 |
| Each dimension is unique and cannot be repeated (no dimension duplication). | 6 |
| No new dimensions or characteristics can be added in the last iteration. | 5, 6 |
| *Subjective ending conditions* |  |
| Concise: the number of dimensions is not un-widely or overwhelming | 6 |
| Robust: enough dimensions and characteristics to classify design techniques | 5, 6 |
| Comprehensive: all design techniques should be classified within the taxonomy | 4, 5, 6 |
| Extendible: a new design technique, characteristic, and dimension can be easily added | 4, 5, 6 |
| Explanatory: the dimensions and characteristics can explain design techniques | 5, 6 |

In the *fifth step*, all the characteristics of the 70 design techniques were gathered. Since not all characteristics were directly presented in the explanation of the sources, the descriptions of each design technique into different characteristics were summarized by applying open coding [8]. The process of open coding was exploratory and can be used to identify concepts. In the coding process, the first coder coded the content, while the second coder checked the coding results. The two coders were the two researchers who evaluated the ending conditions and chose design techniques. For example, if the technique was described that it can be used in a short time period or a couple of days, code “short-term” was given.

The *sixth step* was to group all the characteristics from the fifth step into dimensions. In this step, all the characteristics were summarized into five dimensions. Besides the dimensions that were included in the prior studies, such as design phases, duration, participants, and evaluation types, time dependency (real-time and retrospective) was added as a new dimension. Because real-time and retrospective are two essential characteristics in design processes when identifying design techniques. While real-time describes users’ immediate affective response, retrospective is an indicator of people’s cognitive experience, which can be used to describe a preceding episode of use that happens immediately in the early phase of user test, or a period of days or weeks ago [9,10]. Thus, time dependency needs to be considered as a dimension.

In the *seventh step*, the derived dimensions and characteristics in the taxonomy finally met the criteria of the ending conditions (Table 1). A taxonomy with mutually exclusive and collectively exhaustive dimensions and characteristics was proposed.

1. Taxonomy Evaluation

An overview of the taxonomy evaluation is summarized in Figure 2. In the evaluation, experts with domain knowledge who had not involved in the development of the taxonomy participated in a face-to-face interviews. To structure the interview, an existing framework [11] and appreciative and laddering methods [12] were used. The evaluation was based on the considered design situations in real design projects. The ínterviews were analyzed both qualitatively and quantitatively. In the following, the general evaluation method and the data collection, data analysis, and the results are explained.


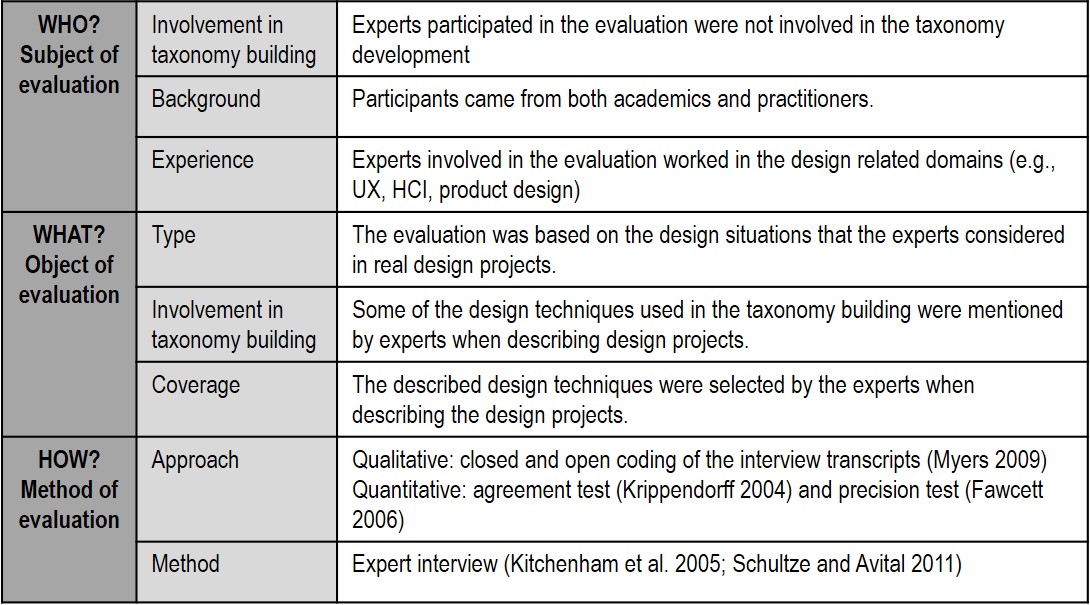


Note: The framework used for summarizing the evaluation approach is adapted from Szopinski et al. (2019).

*Figure 2. An overview of the taxonomy evaluation*

15 experts^[[2]](#footnote-2)^ were interviewed in order to evaluate whether the content of the taxonomy is consistent with the experts’ experiences. Expert interviews provide a source to evaluate whether the content of the taxonomy is understandable and consistent with experts’ working experience [12]. Based on the evaluation purpose, an interview protocol with appreciative questions and laddering questions, the proposed taxonomy, and a questionnaire of demographic information was prepared. All questions were asked in a positive way in order to generate rich data without limiting the experts’ thoughts. As digital service design processes usually need the collaboration of people in the interdisciplinary domain (e.g., interaction design, software engineering), the interviewed experts were from a broad field. Interviews took place face-to-face and were audio recorded after requesting permission from experts. During each interview, additional notes were taken. Each interview took 20-45 minutes. All interviews were transcribed.

In the analysis process, closed coding and open coding were used to evaluate the taxonomy [4]. QDA Miner 5^[[3]](#footnote-3)^, a qualitative data analysis software, was used to analyze the transcripts. First, the interviewer and a second coder who was unfamiliar with the interviews and the taxonomy development process conducted closed coding independently [4]; the codes were the characteristics and dimensions in the taxonomy Krippendorff’s alpha was used to measure the agreement between two coders because of the small sample size of experts [14]. The percentage of the code co-occurrence was 89.8%, and Krippendorff’s alpha was 0.765 (Cohen’s kappa: 0.764; Scott’s pi: 0.765), which was considered as acceptable reliability [14]. The coding frequency results presented that all dimensions and characteristics in the proposed taxonomy were considered to be relevant in the design process.

Second, open coding was conducted by the interviewer to analyze the precision of the proposed taxonomy [15]. Precision means the proportion of positive results in statistics that are true positive results, which is often used in medical diagnoses [15]. In this research, the following formula was applied to calculate precision:

*Precision* = ${\Sigma True positives}/{(\Sigma True positives+\Sigma False positives)}$. (1)

Where *true positives* represent the number of confirmed characteristics from the interviews and *false positives* represent the number of additionally identified characteristics, *precision* means the proportion of the mentioned times of characteristics during the interviews were included in the proposed taxonomy. The large precision (93.78%) indicated that most of the characteristics in the proposed taxonomy were mentioned by experts during interviews.

1. Result

Figure 3 provides a compressed overview of the final version taxonomy for digital service design techniques. Table 2 presents the techniques that assigned into each characteristic.


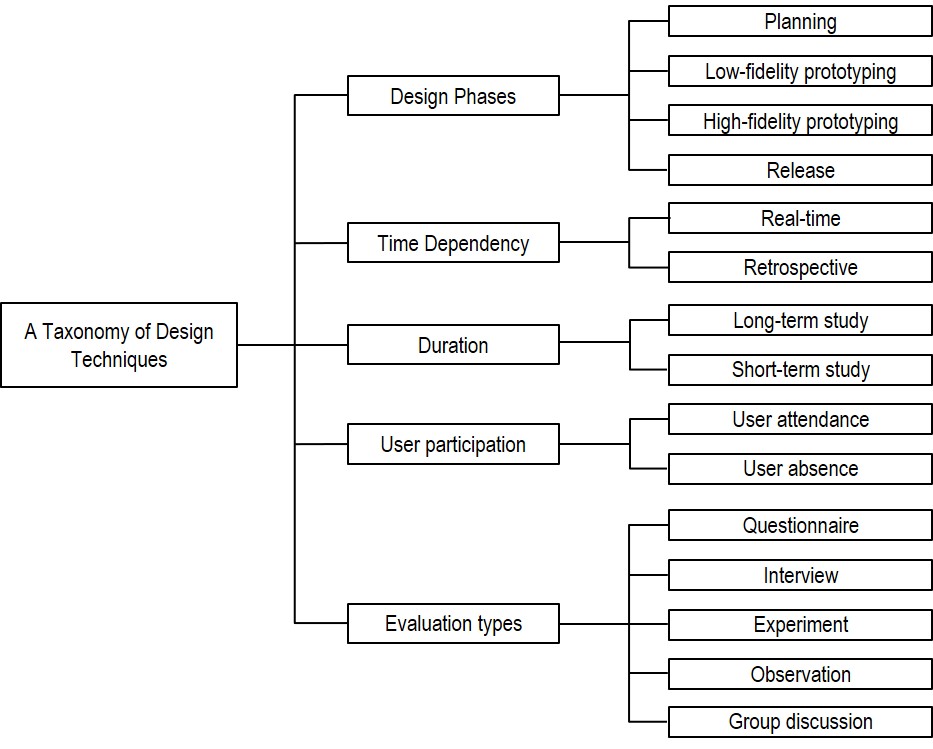


*Figure 3. A taxonomy of digital service design techniques*

*Table 2. A taxonomy of design techniques for digital services*

| **Dimensions and**  **characteristics**  **Design techniques** | ***Design Phases*** | **Planning** | **Low-fidelity prototyping** | **High-fidelity prototyping** | **Release** | ***Time dependency*** | **Real-time** | **Retrospective** | ***Duration*** | **Long-term study** | **Short-term study** | ***User participation*** | **User attendance** | **User absence** | ***Evaluation types*** | **Questionnaire** | **Interview** | **Experiment** | **Observation** | **Group discussion** |
| --- | --- | --- | --- | --- | --- | --- | --- | --- | --- | --- | --- | --- | --- | --- | --- | --- | --- | --- | --- | --- |
| 3-12-3 Brainstorming |  | x |  |  |  |  | x |  |  |  | x |  |  | x |  |  |  |  |  | x |
| 3E (Expressing Experiences and Emotions) |  |  |  | x |  |  |  | x |  |  | x |  | x |  |  | x |  |  |  |  |
| 6-3-5 Brainwriting |  | x |  |  |  |  | x |  |  |  | x |  |  | x |  |  |  |  |  | x |
| A/B Testing |  |  |  | x |  |  | x |  |  |  | x |  | x |  |  |  |  | x |  |  |
| Activity Map |  | x |  |  |  |  | x |  |  |  | x |  |  | x |  |  |  |  |  | x |
| Actors Mapping |  | x |  |  |  |  |  | x |  |  | x |  |  | x |  |  |  |  |  | x |
| Affinity Diagramming |  | x |  |  |  |  |  | x |  |  | x |  |  | x |  |  |  |  |  | x |
| Attribute Listing |  |  |  |  | x |  |  | x |  |  | x |  |  | x |  |  |  |  | x |  |
| Behavioral Mapping |  |  |  |  | x |  | x |  |  | x |  |  | x |  |  |  |  |  | x |  |
| Bodystorming |  |  | x |  |  |  | x |  |  |  | x |  |  | x |  |  |  |  |  | x |
| Business Origami |  |  | x |  |  |  | x |  |  |  | x |  |  | x |  |  |  |  |  | x |
| Co-Discovery |  |  |  | x |  |  | x |  |  |  | x |  | x |  |  |  |  |  | x |  |
| Cognitive Mapping |  | x |  |  |  |  | x |  |  |  | x |  |  | x |  |  |  |  |  | x |
| Cognitive Walkthrough |  |  | x |  |  |  | x |  |  |  | x |  |  | x |  |  | x |  |  |  |
| Collaborative Sketching |  |  | x |  |  |  | x |  |  |  | x |  |  | x |  |  |  |  |  | x |
| Concept Mapping |  | x |  |  |  |  | x |  |  |  | x |  | x |  |  |  |  |  |  | x |
| Concurrent Think-Aloud |  |  |  | x |  |  | x |  |  |  | x |  | x |  |  |  |  |  | x |  |
| Content Inventory & Audit |  |  |  |  | x |  |  | x |  |  | x |  |  | x |  |  |  |  | x |  |
| Contextual Laddering |  | x |  |  |  |  | x |  |  |  | x |  | x |  |  |  | x |  |  |  |
| Critical Incident Technique |  |  |  |  | x |  | x |  |  |  | x |  | x |  |  |  | x |  |  |  |
| Desirability Testing with Product Reaction Cards |  |  | x |  |  |  | x |  |  |  | x |  | x |  |  |  |  |  |  | x |
| Diary Studies |  |  |  |  | x |  |  | x |  | x |  |  | x |  |  | x |  |  |  |  |
| Directed Storytelling |  | x |  |  |  |  | x |  |  |  | x |  |  | x |  |  |  |  |  | x |
| Experience Clips |  |  |  |  | x |  | x |  |  |  | x |  | x |  |  |  |  |  | x |  |
| Experience Prototyping |  |  | x |  |  |  | x |  |  |  | x |  |  | x |  |  |  |  | x |  |
| Eye-tracking |  |  |  | x |  |  | x |  |  |  | x |  | x |  |  |  |  | x |  |  |
| Flexible Modeling |  |  | x |  |  |  | x |  |  |  | x |  | x |  |  |  |  |  |  | x |
| Fly-on-the-Wall Observation |  | x |  |  |  |  | x |  |  |  | x |  | x |  |  |  |  |  | x |  |
| Graffiti Walls |  | x |  |  |  |  | x |  |  | x |  |  | x |  |  |  |  |  | x |  |
| Greeting Cards |  | x |  |  |  |  | x |  |  |  | x |  |  | x |  |  |  |  |  | x |
| Heuristic Evaluation |  |  | x |  |  |  | x |  |  |  | x |  |  | x |  | x |  |  |  |  |
| Kano Analysis |  | x |  |  |  |  | x |  |  |  | x |  | x |  |  | x |  |  |  |  |
| LEGO Serious Play |  | x |  |  |  |  | x |  |  |  | x |  |  | x |  |  |  |  |  | x |
| Mental Model Diagramming |  | x |  |  |  |  |  | x |  | x |  |  | x |  |  |  |  |  |  | x |
| Mind Mapping |  | x |  |  |  |  |  | x |  |  | x |  |  | x |  |  |  |  |  | x |
| Mood Boards |  |  | x |  |  |  |  | x |  |  | x |  |  | x |  |  |  |  |  | x |
| Motivation Matrix |  | x |  |  |  |  |  | x |  |  | x |  |  | x |  | x |  |  |  |  |
| Offering Mapping |  | x |  |  |  |  |  | x |  |  | x |  |  | x |  |  |  |  |  | x |
| Parallel prototyping |  |  | x |  |  |  | x |  |  | x |  |  |  | x |  |  |  |  |  | x |
| Personas |  | x |  |  |  |  |  | x |  |  | x |  |  | x |  |  |  |  |  | x |
| Phillips 66 Method |  | x |  |  |  |  | x |  |  |  | x |  |  | x |  |  |  |  |  | x |
| Photo Diary |  |  |  |  | x |  | x |  |  | x |  |  | x |  |  |  |  |  | x |  |
| Photo Elicitation Interviewing |  | x |  |  |  |  |  | x |  | x |  |  | x |  |  |  | x |  |  |  |
| Private Camera Conversation |  |  |  | x |  |  |  | x |  |  | x |  | x |  |  |  | x |  |  |  |
| Product Experience Tracker |  |  |  |  | x |  |  | x |  | x |  |  | x |  |  | x |  |  |  |  |
| Repertory Grids |  |  | x |  |  |  |  | x |  |  | x |  | x |  |  |  |  | x |  |  |
| Retrospective Think-Aloud |  |  |  | x |  |  |  | x |  |  | x |  | x |  |  |  |  |  | x |  |
| Role-Playing |  | x |  |  |  |  | x |  |  |  | x |  |  | x |  |  |  |  |  | x |
| Scenarios |  | x |  |  |  |  |  | x |  |  | x |  |  | x |  |  |  |  |  | x |
| Service Blueprints |  | x |  |  |  |  |  | x |  |  | x |  | x |  |  |  |  |  |  | x |
| Shadowing |  | x |  |  |  |  | x |  |  |  | x |  | x |  |  |  |  |  | x |  |
| Speed Dating |  |  | x |  |  |  | x |  |  |  | x |  | x |  |  |  |  |  | x |  |
| Stakeholder Walkthrough |  |  | x |  |  |  | x |  |  |  | x |  | x |  |  |  |  |  |  | x |
| Story Sharing |  | x |  |  |  |  | x |  |  |  | x |  |  | x |  |  |  |  |  | x |
| Storyboarding |  | x |  |  |  |  |  | x |  |  | x |  |  | x |  |  |  |  |  | x |
| Sustainability Map |  | x |  |  |  |  | x |  |  |  | x |  |  | x |  |  |  |  |  | x |
| Teachback |  |  | x |  |  |  | x |  |  |  | x |  | x |  |  |  | x |  |  |  |
| Territory Maps |  | x |  |  |  |  | x |  |  |  | x |  |  | x |  |  |  |  |  | x |
| The Love Letter & the Breakdown Letter |  | x |  |  |  |  |  | x |  |  | x |  | x |  |  | x |  |  |  |  |
| Thought Leader |  | x |  |  |  |  | x |  |  |  | x |  |  | x |  |  |  |  |  | x |
| Time Machine |  | x |  |  |  |  | x |  |  |  | x |  |  | x |  |  |  |  |  | x |
| Tomorrow Headlines |  | x |  |  |  |  | x |  |  |  | x |  |  | x |  |  |  |  |  | x |
| Touchpoint Matrix |  | x |  |  |  |  |  | x |  |  | x |  |  | x |  |  |  |  | x |  |
| Trigger |  | x |  |  |  |  | x |  |  |  | x |  |  | x |  |  |  |  |  | x |
| User Journey Maps |  |  | x |  |  |  |  | x |  |  | x |  | x |  |  |  |  |  | x |  |
| UX Curve |  |  |  |  | x |  |  | x |  | x |  |  | x |  |  | x |  |  |  |  |
| Web Analytics |  |  |  |  | x |  | x |  |  | x |  |  | x |  |  |  |  |  | x |  |
| Wireframing |  |  | x |  |  |  |  | x |  |  | x |  |  | x |  |  |  |  |  | x |
| Wishful Thinking |  | x |  |  |  |  | x |  |  |  | x |  |  | x |  |  |  |  |  | x |
| Wizard of Oz |  |  | x |  |  |  | x |  |  |  | x |  | x |  |  |  |  | x |  |  |
| ***Note: 40 design techniques from this table were used in the experiment.*** | | | | | | | | | | | | | | | | | | | | |

Reference

[1] R.C. Nickerson, U. Varshney, J. Muntermann, A method for taxonomy development and its application in information systems, Eur. J. Inf. Syst. 22 (2013) 336–359. https://doi.org/10.1057/ejis.2012.26.

[2] E. Boulton, H. Hawley-Hague, B. Vereijken, A. Clifford, N. Guldemond, K. Pfeiffer, A. Hall, F. Chesani, S. Mellone, A. Bourke, C. Todd, Developing the FARSEEING Taxonomy of Technologies: Classification and description of technology use (including ICT) in falls prevention studies, J. Biomed. Inform. 61 (2016) 132–140. https://doi.org/10.1016/j.jbi.2016.03.017.

[3] N. Prat, I. Comyn-Wattiau, J. Akoka, A Taxonomy of Evaluation Methods for Information Systems Artifacts, J. Manag. Inf. Syst. 32 (2015) 229–267. https://doi.org/10.1080/07421222.2015.1099390.

[4] M.D. Myers, Qualitative Research in Business & Management, 1st ed., Sage, London, 2009.

[5] B. Mager, Service Design as an Emerging Field, in: S. Miettinen, M. Koivisto (Eds.), Des. Serv. with Innov. Methods, University of Art and Design Helsinki, 2009, pp. 28–43.

[6] K. Williams, S. Chatterjee, M. Rossi, Design of emerging digital services: a taxonomy, Eur. J. Inf. Syst. 17 (2008) 505–517. https://doi.org/10.1057/ejis.2008.38.

[7] K. Kutsikos, N. Konstantopoulos, D. Sakas, Y. Verginadis, Developing and managing digital service ecosystems: a service science viewpoint, J. Syst. Inf. Technol. 16 (2014) 233–248. https://doi.org/10.1108/JSIT-02-2014-0015.

[8] J. Corbin, A. Strauss, Basics of Qualitative Research: Techniques and Procedures for Developing Grounded Theory, Fourth, Sage, Thousand Oaks, CA, 2014.

[9] J.H. Kim, D. V Gunn, E. Schuh, B. Phillips, R.J. Pagulayan, D. Wixon, Tracking real-time user experience (TRUE): A comprehensive instrumentation solution for complex systems, in: Proceeding Twenty-Sixth Annu. CHI Conf. Hum. Factors Comput. Syst. - CHI ’08, ACM Press, Florence, Italy, 2008, pp. 443–451https://doi.org/10.1145/1357054.1357126.

[10] M. Hassenzahl, D. Ullrich, To do or not to do: Differences in user experience and retrospective judgments depending on the presence or absence of instrumental goals, Interact. Comput. 19 (2007) 429–437. https://doi.org/10.1016/j.intcom.2007.05.001.

[11] B. Kitchenham, S. Linkman, S. Linkman, Experiences of using an evaluation framework, Inf. Softw. Technol. 47 (2005) 761–774. https://doi.org/10.1016/j.infsof.2005.01.001.

[12] U. Schultze, M. Avital, Designing interviews to generate rich data for information systems research, Inf. Organ. 21 (2011) 1–16. https://doi.org/10.1016/j.infoandorg.2010.11.001.

[13] D. Szopinski, T. Schoormann, D. Kundisch, Because your taxonomy is worth it: Towards a framework for taxonomy evaluation, in: Proc. 27th Eur. Conf. Inf. Syst., Stockholm & Uppsala, Sweden, 2019, pp. 1–20. https://aisel.aisnet.org/ecis2019_rp/104.

[14] K. Krippendorff, Content Analysis: An Introduction to Its Methodology, Second, Sage, Thousand Oaks, CA, 2004.

[15] T. Fawcett, An introduction to ROC analysis, Pattern Recognit. Lett. 27 (2006) 861–874. https://doi.org/10.1016/j.patrec.2005.10.010.

[16] A.P.O.S. Vermeeren, E.L.-C. Law, V. Roto, M. Obrist, J. Hoonhout, K. Väänänen-Vainio-Mattila, User experience evaluation methods: Current state and development needs, in: Proc. 6th Nord. Conf. Human-Computer Interact. Extending Boundaries - Nord. ’10, ACM Press, Reykjavik, Iceland, 2010, pp. 521–530https://doi.org/10.1145/1868914.1868973.

[17] R. Curedale, Service Design: 250 essential methods, Design Community College Inc., Topanga CA, 2013.

[18] M. Vigo, S. Bail, C. Jay, R. Stevens, Overcoming the pitfalls of ontology authoring: Strategies and implications for tool design, Int. J. Hum. Comput. Stud. 72 (2014) 835–845. https://doi.org/10.1016/j.ijhcs.2014.07.005.

[19] N. Dell, N. Kumar, The Ins and Outs of HCI for Development, in: Proc. 2016 CHI Conf. Hum. Factors Comput. Syst. - CHI ’16, ACM Press, San Jose, CA, 2016, pp. 2220–2232https://doi.org/10.1145/2858036.2858081.

1. i) [*usability.gov*](http://www.usability.gov/) is a leading resource for UX practices and introduces 32 design and evaluation methods; ii) [*servicedesigntools.org*](http://www.servicedesigntools.org/) is a research project conducted Research & Consulting Center of Domus Academy, which contains 36 methods, techniques and tools for service design; iii) *allaboutux.org* is the result of a survey conducted by Vermeeren et al. (2010) that provides a list of 82 evaluation methods for UX; iv) *Service Design* is a book that includes 250 methods, techniques, and tools for service desing [17]; v) *Universal Methods of Design* is a book that includes 100 methods and techniques for widespread use in product and service design (Martin and Hanington 2012). [↑](#footnote-ref-1)
2. 15 experts were considered to be sufficient to evaluate the proposed taxonomy (cf. Dell and Kumar 2016; Vigo et al. 2014). [↑](#footnote-ref-2)
3. https://provalisresearch.com/products/qualitative-data-analysis-software/ [↑](#footnote-ref-3)
